# The association between a history of anxiety or depression and utilization of diagnostic imaging

**DOI:** 10.1371/journal.pone.0254572

**Published:** 2021-07-12

**Authors:** Adam C. Powell, James W. Long, Garry Carneal, Kathryn J. Schormann, David P. Friedman

**Affiliations:** 1 HealthHelp, Houston, TX, United States of America; 2 Humana Inc., Louisville, KY, United States of America; 3 The Kennedy Forum, Annapolis, MD, United States of America; 4 Thomas Jefferson University, Philadelphia, PA, United States of America; Maastricht University Medical Center, NETHERLANDS

## Abstract

**Objective:**

While prior research shows that mental illness is associated with lower utilization of screening imaging, little is known about how mental illness impacts use of diagnostic imaging, other than for screening. This study explores the association between a history of anxiety or depression in the prior year and utilization of diagnostic imaging.

**Methods:**

Commercial and Medicare Advantage health plan claims from 2017 and 2018 from patients with plans from one national organization were extracted. Exclusions were made for patients without continuous plan enrollment. History of anxiety or depression was determined using 2017 claims, and downstream diagnostic imaging was determined using 2018 claims. Univariate associations were assessed with Chi-square tests. A matched sample was created using Coarsened Exact Matching, with history of mental illness serving as the treatment variable. Logistic regressions were used to calculate adjusted odds ratios, before and after matching, controlling for age, sex, urbanicity, local income, comorbidities, claims history, region, and health plan characteristics. Associations between mental illness and chest imaging, neuroimaging, and emergency department imaging were also evaluated.

**Results:**

The sample included 2,381,851 patients before matching. Imaging was significantly more likely for patients with a history of anxiety (71.1% vs. 55.7%, *P* < .001) and depression (73.2% vs. 55.3%, *P* < .001). The adjusted odds of any imaging were 1.24 (95% confidence interval [CI]: 1.22–1.26) for patients with a history of anxiety, and 1.43 (CI: 1.41–1.45) for patients with a history of depression before matching, and 1.18 (CI: 1.16–1.20) for a history of anxiety and 1.33 (CI: 1.32–1.35) for a history of depression after matching. Adjusted analyses found significant, positive associations between mental illness and chest imaging, neuroimaging, and emergency department imaging both before and after matching.

**Discussion:**

In contrast to prior findings on screening, anxiety and depression were associated with greater likelihood of diagnostic imaging within the population studied.

## Introduction

Health equity has been a pressing concern of many organizations, including the World Health Organization, the American Academy of Family Physicians, as well as in the radiology community [1–3]. Health equity is realized when everyone has the opportunity to achieve their best potential health. Mental illness has been shown to be a source of inequity in access to care related to physical health. For example, prior research has suggested that people with a history of mental illness are less able to access various types of screening imaging. Anxiety and depression have been shown to be risk factors for the underuse of screening mammography [4–7]. Mental illness has likewise been shown to be a barrier to lung cancer screening by low-dose computed tomography [8]. A meta-analysis concluded that screening for any cancer has been found to occur significantly less frequently in people with any mental illness than in the general population [9].

Little is known regarding whether mental illness is associated with reduced utilization of diagnostic imaging, and the extent to which an inequity exists within a privately insured population. While screening imaging is performed on people with no suspicion of a disease, and is somewhat elective in nature, diagnostic imaging is performed in response to clinical suspicion of a disease and is less elective. For instance, it is recommended that some people with a history of smoking receive a screening via computed tomography (CT), even if they have no signs of cancer [10]. In contrast, a patient with Pancoast syndrome would receive a diagnostic lung CT. Given the difference in the circumstances under which diagnostic versus screening imaging is performed, it is possible that anxiety and depression impact diagnostic imaging differently than they impact screening. To the extent that psychosomatic symptoms present in patients with anxiety and depression, these illnesses may increase diagnostic imaging utilization, rather than reduce it. Alternatively, anxiety and depression could potentially reduce diagnostic imaging utilization if they make people less willing to leave their homes to seek diagnosis, or impact peoples’ willingness to undergo diagnostic imaging.

The purpose of this study is to assess whether anxiety and depression are associated with a diagnostic imaging utilization disparity, analogous to the disparity that has been demonstrated in the context of screening imaging. The association between a history of anxiety or depression and chest imaging, neuroimaging, and diagnostic imaging occurring in an emergency department (ED) setting are each explored separately to help better characterize the nature of the imaging received. As anxiety and depression can cause people to experience chest pain, it is possible that they lead to elevated levels of chest imaging and imaging in an ED setting [11]. Since patients with anxiety or depression may complain of headaches or other forms of brain-based somatic illness, and signs of these mental illnesses may be physically observable in the brain, the analysis separately examined whether a history of mental illness has a differential impact on neuroimaging [12, 13]. Finally, as social support and imaging needs vary across the course of a person’s life, additional age-stratified analyses were conducted to characterize how age moderates the relationship between mental illness and diagnostic imaging utilization.

## Materials and methods

### Data source and sample population

Commercial (privatized, employer-sponsored) and Medicare Advantage (privatized, government-sponsored) health plan claims from calendar years 2017 and 2018, pertaining to patients with health plans from one national healthcare organization, were extracted. Patients were included in the sample if they had a health plan from the organization in 2017 that contractually allowed participation in research. Patients were excluded from the sample if they were not continuously enrolled in their health plan from January 1^st^ 2017 to December 31^st^ 2018, or if they were not between ages 18 and 89. Finally, patients were excluded if it was not possible to link them to 2017 comorbidity flags that had been generated by the national healthcare organization independently from this analysis.

In addition to the sample of patients constructed before matching, two additional matched samples were constructed. Coarsened Exact Matching (CEM) was used to construct a sample in which patients were matched on covariates, with a history of mental illness (anxiety or depression) serving as the treatment variable [14]. As racial data was only available for patients with Medicare Advantage plans, patients with commercial health plans were excluded from the sample constructed before matching, and a second matched sample of patients with Medicare Advantage health plans was constructed using CEM. The second sample matched patients on race, as well as the covariates considered in constructing the first sample.

This study was reviewed by the Advarra institutional review board (Pro00036954), and on June 25, 2019, received an exemption from oversight in accordance with the Department of Health and Human Services regulations found in Title 45 of the Code of Federal Regulations. The study was conducted in accordance with the Declaration of Helsinki.

### Measurement

The independent variables in the analyses were whether the patient had a history of anxiety or depression in calendar year 2017 medical claims, as indicated by the International Classification of Diseases version 9 or 10 codes associated with the claims. All the diagnosis coding definitions used in this study are provided in Appendix A in S1 File. Patients with no claims in 2017 were considered to not have had a history of anxiety or depression. Claims including codes for illnesses related to anxiety or depression could come from any healthcare provider; claims from internists, family physicians, and other healthcare providers outside of the core of the behavioral health system were also considered.

For each patient, the total number of outpatient diagnostic imaging claims in calendar year 2018 were counted. Claims were grouped by Healthcare Common Procedure Coding System code according to the scheme shown in Appendix B in S1 File. Diagnostic imaging claims consisted of claims for computed tomography, magnetic resonance imaging, positron emission tomography, plain film radiography, ultrasound, and other modalities. Codes were selected in consultation with a board-certified academic radiologist specialized in neuroimaging and a medical coding expert. While these counts served as one dependent variable, four other dependent variables were derived from the claims that were counted: a binary variable indicating whether each patient received any imaging, a binary variable indicating whether each patient received any chest imaging, a binary variable indicating whether each patient received any neuroimaging, and a binary variable indicating whether each patient received any imaging in an ED setting.

### Outcomes and analysis

All statistical analyses were conducted using R version 4.0.3, from the R Foundation for Statistical Computing. Descriptive statistics were calculated for the overall population in the sample constructed before matching, with a breakout of individuals with and without a history of mental illness (anxiety or depression). Descriptive statistics were additionally calculated for the matched sample (not considering race), and for the matched sample of patients with Medicare Advantage health plans in which race served as a covariate.

Using Chi-square tests, the univariate associations between utilization of any imaging and a history of anxiety, as well as a history of depression, were calculated. These univariate analyses were repeated with use of any chest imaging, neuroimaging, and ED imaging serving as dependent variables.

To control for other factors that may have influenced imaging utilization, a series of multivariate logistic regressions were run, with 2018 claims-based measures of any imaging, any chest imaging, any neuroimaging, and any imaging in an ED setting serving as dependent variables of the models. History of anxiety and history of depression in 2017 served as the independent variables of the regressions, and patient age, urbanicity (rural versus urban), health plan line of business (commercial versus Medicare Advantage), health plan type (fee-for-service versus health maintenance organization versus preferred provider organization), sex, and residency in a ZIP code with median income below $40,000, diagnosis code-based history of comorbidities (coronary artery disease, congestive heart failure, chronic obstructive pulmonary disease), claims-based history of prior procedures (imaging, cardiology, oncology), prevalence of obesity in the patient’s state, and patient’s region serving as the control variables. In one analysis, race was additionally used as a control variable. The urbanicity of patients’ ZIP codes were determined using a mapping table from the Centers for Medicare & Medicaid Services (CMS) [15]. The median income within patients’ ZIP Codes were determined using data from American Community Survey’s 2013–2017 5-year estimates of median income, reporting income in 2017 inflation-adjusted dollars [16]. The average rate of obesity in patients’ home states was determined using 2018 data from the Behavioral Risk Factor Surveillance Survey released by the Centers for Disease Control and Prevention [17]. Region was assigned using the methodology used by CMS [18]. Results from the regressions were reported as odds ratios (ORs) with 95% confidence intervals (CIs).

Since people of varying ages have differing degrees of social support in obtaining needed medical care, and older people on average require a greater quantity of imaging, additional analyses were performed to evaluate the role of age in the relationship between mental illness and diagnostic imaging utilization [19]. The adjusted analysis was repeated for different age strata: ages 18 to 29, 30 to 44, 45 to 64, and 65 or older. To quantify the imaging performed for each age strata and clinical presentation (all, anxiety, no anxiety, depression, no depression), the percentage of patients receiving images was calculated for each age/presentation combination. Secondly, the mean number of images received by patients in each age strata was also reported. Within each clinical presentation category, the significance of the association between age and utilization of imaging was assessed using a logistic regression, and the significance of the association between age and quantity of imaging, conditional upon a patient having received any imaging, was assessed using a Poisson regression. Finally, the adjusted association between a history of anxiety, a history of depression, and the quantity of imaging utilization was calculated using a Poisson regression.

To account for potential differences between people with a history of mental illness and people without a history of mental illness, the multivariate logistic regressions were each recomputed using a matched sample constructed using CEM, where a history of mental illness (anxiety or depression) served as the treatment variable. The multivariate logistic regressions were run an additional time with a matched sample of patients with Medicare Advantage health plans, with race included as a covariate. Results were again reported as ORs with 95% CIs.

## Results

**Fig 1:**
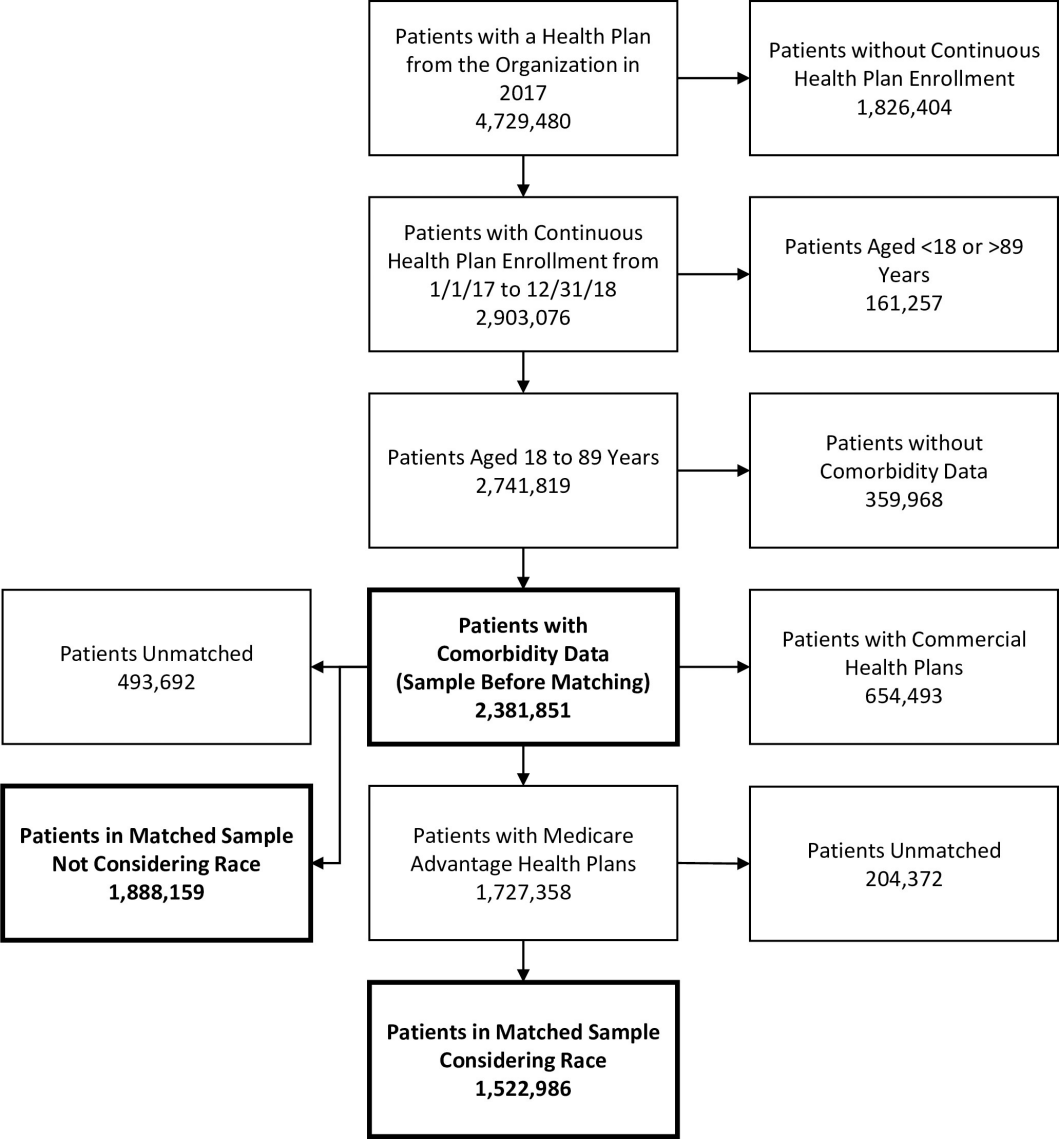
Participant flow diagram.

As shown in Fig 1, of the 4,729,480 patients that met initial inclusion criteria, 2,381,851 remained after exclusion criteria were applied. Using patients from this population, a matched sample not considering race was formed using a subpopulation of 1,888,159 patients, and a second matched sample considering race was formed using a subpopulation of 1,522,986 patients with Medicare Advantage health plans. Within the original sample of 2,381,851 patients, 83,146 patients had a history of anxiety, 124,101 patients had a history of depression, and 19,884 patients had a history of both.

As described in Table 1, patients with a history of mental illness were significantly younger, more likely to live in rural areas, to have a preferred provider organization health plan, and to be female, to have a history of cardiology claims, to have a history of imaging claims, to have congestive heart failure, and to have chronic obstructive pulmonary disease, and significantly less likely to have a history of oncology claims, coronary artery disease, or diabetes. The healthcare organization offering the health plans had a heavy Medicare Advantage emphasis, with 90.9% of included patients holding Medicare Advantage plans. Consequently, the mean age of included patients was 70.3 years. Patients predominantly resided in CMS Region 4; an area consisting of Alabama, Florida, Georgia, Kentucky, Mississippi, North Carolina, South Carolina, and Tennessee.

**Table 1:** Descriptive statistics.

	All (N = 2,381,851)	No Mental Illness (n = 2,194,488)	Mental Illness (n = 187,363)	P-Value	No Mental Illness, Matched (n = 1,713,650)	Mental Illness, Matched (n = 174,509)	*P*-Value (considering weights)	No Mental Illness, Matched with Race (n = 1,370,437)	Mental Illness, Matched with Race (n = 152,549)	*P*-Value (considering weights)
Age, years	70.31	70.71	65.60	< .001	70.59	66.18	0.606	72.84	68.64	0.606
Rural, *n (%)*	471,799 (19.8%)	428,063 (19.5%)	43,736 (23.3%)	< .001	274,375 (16.0%)	38,902 (22.3%)	1.000	239,042 (17.4%)	36,000 (23.6%)	1.000
Commercial, *n (%)*	216,190 (9.1%)	199,156 (9.1%)	17,034 (9.1%)	0.819	144,875 (8.5%)	15,926 (9.1%)	1.000	-	-	-
Plan Type										
*FFS*, *n (%)*	67,944 (2.9%)	199,156 (9.1%)	7,015 (3.7%)	< .001	23,783 (1.4%)	5,116 (2.9%)	1.000	19,758 (1.4%)	4,700 (3.1%)	1.000
*HMO*, *n (%)*	1,198,341 (50.3%)	1,117,480 (50.9%)	80,861 (43.2%)		887,213 (51.8%)	75,849 (43.5%)		663,143 (48.4%)	62,442 (40.9%)	
*PPO*, *n (%)*	1,115,566 (46.8%)	1,016,079 (46.3%)	99,487 (53.1%)		802,654 (46.8%)	93,544 (53.6%)		687,536 (50.2%)	85,407 (56.0%)	
Female, *n (%)*	1,318,482 (55.4%)	1,188,904 (54.2%)	129,578 (69.2%)	< .001	1,000,813 (58.4%)	121,390 (69.6%)	1.000	820,821 (59.9%)	106,631 (69.9%)	1.000
Below $40k Income, *n (%)*	566,452 (23.8%)	521,817 (23.8%)	44,635 (23.8%)	0.702	351,165 (20.5%)	40,140 (23.0%)	0.331	272,843 (19.9%)	36,012 (23.6%)	0.359
2017 Cardiology Claims, *n (%)*	1,663,690 (69.8%)	1,508,377 (68.7%)	155,313 (82.9%)	< .001	1,197,327 (69.9%)	145,356 (83.3%)	1.000	1,009,915 (73.7%)	131,983 (86.5%)	1.000
2017 Imaging Claims, *n (%)*	1,346,477 (56.5%)	1,207,676 (55.0%)	138,801 (74.1%)	< .001	974,065 (56.8%)	129,664 (74.3%)	1.000	808,231 (59.0%)	115,947 (76.0%)	1.000
2017 Oncology Claims, *n (%)*	47,243 (2.0%)	43,659 (2.0%)	3,584 (1.9%)	0.023	11,344 (0.7%)	2,231 (1.3%)	1.000	8,649 (0.6%)	1,855 (1.2%)	1.000
2017 CAD, *n (%)*	533,535 (22.4%)	493,427 (22.5%)	40,108 (21.4%)	< .001	326,845 (19.1%)	35,653 (20.4%)	1.000	274,494 (20.0%)	33,338 (21.9%)	1.000
2017 CHF, *n (%)*	242,571 (10.2%)	222,608 (10.1%)	19,963 (10.7%)	< .001	115,277 (6.7%)	16,544 (9.5%)	1.000	90,467 (6.6%)	15,146 (9.9%)	1.000
2017 COPD, *n (%)*	348,781 (14.6%)	311,857 (14.2%)	36,924 (19.7%)	< .001	193,919 (11.3%)	31,688 (18.2%)	1.000	164,993 (12.0%)	29,649 (19.4%)	1.000
2017 Diabetes, *n (%)*	789,478 (33.1%)	734,535 (33.5%)	54,943 (29.3%)	< .001	519,734 (30.3%)	49,779 (28.5%)	1.000	416,695 (30.4%)	45,818 (30.0%)	1.000
Prevalence of Obesity	33.1%	33.1%	33.7%	< .001	33.2%	33.7%	0.999	33.3%	33.7%	0.988
Region										
* # in Region 1*, *n (%)*	7,096 (0.3%)	6,177 (0.3%)	919 (0.5%)	< .001	2,050 (0.1%)	596 (0.3%)	1.000	1,865 (0.1%)	590 (0.4%)	1.000
*# in Region 2*, *n (%)*	12,663 (0.5%)	11,459 (0.5%)	1,204 (0.6%)		5,839 (0.3%)	959 (0.5%)		4,285 (0.3%)	860 (0.6%)	
*# in Region 3*, *n (%)*	184,313 (7.7%)	166,460 (7.6%)	17,853 (9.5%)		117,571 (6.9%)	16,200 (9.3%)		101,362 (7.4%)	15,543 (10.2%)	
*# in Region 4*, *n (%)*	991,407 (41.6%)	921,836 (42.0%)	69,571 (37.1%)		750,586 (43.8%)	65,612 (37.6%)		583,703 (42.6%)	55,547 (36.4%)	
*# in Region 5*, *n (%)*	370,611 (15.6%)	335,697 (15.3%)	34,914 (18.6%)		271,214 (15.8%)	32,886 (18.8%)		224,646 (16.4%)	28,989 (19.0%)	
*# in Region 6*, *n (%)*	523,103 (22.0%)	480,390 (21.9%)	42,713 (22.8%)		402,930 (23.5%)	40,777 (23.4%)		327,505 (23.9%)	35,844 (23.5%)	
*# in Region 7*, *n (%)*	85,377 (3.6%)	77,206 (3.5%)	8,171 (4.4%)		49,711 (2.9%)	7,092 (4.1%)		37,891 (2.8%)	5,844 (3.8%)	
*# in Region 8*, *n (%)*	42,960 (1.8%)	38,548 (1.8%)	4,412 (2.4%)		21,301 (1.2%)	3,537 (2.0%)		17,781 (1.3%)	3,125 (2.0%)	
*# in Region 9*, *n (%)*	137,387 (5.8%)	132,386 (6.0%)	5,001 (2.7%)		76,116 (4.4%)	4,562 (2.6%)		57,494 (4.2%)	4,052 (2.7%)	
*# in Region 10*, *n (%)*	26,934 (1.1%)	24,329 (1.1%)	2,605 (1.4%)		16,332 (1.0%)	2,288 (1.3%)		13,905 (1.0%)	2,155 (1.4%)	
2018 Any Imaging, n (%)	1,338,504 (56.2%)	1,203,658 (54.8%)	134,846 (72.0%)	< .001	931,920 (54.4%)	125,181 (71.7%)	< .001	769,504 (56.2%)	112,137 (73.5%)	< .001
2018 Chest Imaging, n (%)	141,631 (5.9%)	127,984 (5.8%)	13,647 (7.3%)	< .001	95,510 (5.6%)	12,549 (7.2%)	< .001	81,501 (5.9%)	11,705 (7.7%)	< .001
2018 Neuroimaging, n (%)	251,551 (10.6%)	216,191 (9.9%)	35,360 (18.9%)	< .001	161,079 (9.4%)	32,582 (18.7%)	< .001	136,905 (10.0%)	30,128 (19.7%)	< .001
2018 ED Imaging, n (%)	483,457 (20.3%)	421,972 (19.2%)	61,485 (32.8%)	< .001	309,741 (18.1%)	56,070 (32.1%)	< .001	257,661 (18.8%)	51,120 (33.5%)	< .001
Race										
*Race Unknown*, *n (%)*	-	-	-	-	-	-	-	5,899 (0.4%)	764 (0.5%)	1.000
*White*, *n (%)*	-	-	-		-	-		1,218,985 (88.9%)	137,273 (90.0%)	
*Black*, *n (%)*	-	-	-		-	-		124,553 (9.1%)	12,367 (8.1%)	
*Other*, *n (%)*	-	-	-		-	-		11,651 (0.9%)	973 (0.6%)	
*Asian*, *n (%)*	-	-	-		-	-		2,404 (0.2%)	254 (0.2%)	
*Hispanic*, *n (%)*	-	-	-		-	-		6,719 (0.5%)	798 (0.5%)	
*Native American*, *n (%)*	-	-	-		-	-		226 (0.0%)	120 (0.1%)	

*Abbreviations*: *Coronary Artery Disease (CAD); Chronic Obstructive Pulmonary Disease (COPD); Congestive Heart Failure (CHF); Emergency Department (ED); Fee-For-Service (FFS); Health Maintenance Organization (HMO); Preferred Provider Organization (PPO)*

### Findings from the sample before matching

Univariate analyses found that patients with a history of anxiety were significantly more likely to use imaging (71.1% [59,100/83,146] vs. 55.7% [1,279,404/2,298,705], *P*<0.001). Patients with a history of depression were likewise significantly more likely to use imaging (73.2% [90,846/124,101] vs. 55.3% [1,247,658/2,257,750], *P*<0.001). A multivariate analysis of the sample before matching (Table 2) found that when considered concurrently, along with control variables, a history of anxiety was associated with increased odds of any imaging (1.24; 95% CI: 1.22–1.26), as was a history of depression (1.43; 95% CI: 1.41–1.45).

**Table 2:** Adjusted odds ratios for factors influencing use of any imaging (sample before matching).

	Any Imaging		Chest Imaging		Neuroimaging Imaging		ED Imaging	
**Variable**	**OR**	**95% CI**	**OR**	**95% CI**	**OR**	**95% CI**	**OR**	**95% CI**
Anxiety	1.24	1.22–1.26	1.06	1.03–1.09	1.48	1.45–1.51	1.46	1.43–1.48
Depression	1.43	1.41–1.45	1.08	1.06–1.11	1.83	1.80–1.86	1.55	1.53–1.57
Age	1.00	1.00–1.00	1.00	1.00–1.00	1.01	1.01–1.01	1.00	1.00–1.00
Rural (as opposed to Urban)	0.99	0.98–1.00	0.96	0.94–0.97	0.96	0.95–0.97	1.07	1.06–1.08
Commercial (as opposed to Medicare)	1.07	1.06–1.09	0.77	0.75–0.80	0.81	0.79–0.83	0.76	0.75–0.77
Plan Type: HMO (as opposed to FFS)	0.72	0.70–0.73	0.87	0.84–0.90	0.83	0.81–0.85	0.86	0.85–0.88
Plan Type: PPO (as opposed to FFS)	1.09	1.07–1.11	1.13	1.09–1.16	0.96	0.94–0.98	0.97	0.96–0.99
Female	1.40	1.39–1.41	0.91	0.90–0.92	1.15	1.14–1.16	1.14	1.13–1.15
Below $40k Income	0.96	0.95–0.97	1.00	0.98–1.01	1.02	1.01–1.03	1.09	1.08–1.10
2017 Cardiology Claims	3.31	3.29–3.34	3.11	3.04–3.18	2.26	2.22–2.29	2.27	2.25–2.30
2017 Imaging Claims	3.24	3.22–3.26	1.66	1.63–1.68	2.11	2.09–2.13	2.17	2.15–2.18
2017 Oncology Claims	1.86	1.81–1.91	0.99	0.96–1.02	1.45	1.41–1.48	1.25	1.22–1.27
2017 CAD	1.21	1.20–1.22	2.54	2.51–2.57	1.27	1.26–1.28	1.35	1.34–1.36
2017 CHF	1.16	1.14–1.17	1.14	1.12–1.16	1.37	1.35–1.39	1.62	1.60–1.63
2017 COPD	1.32	1.31–1.34	1.13	1.12–1.15	1.23	1.22–1.25	1.56	1.54–1.57
2017 Diabetes	1.02	1.02–1.03	1.25	1.23–1.26	1.20	1.19–1.21	1.24	1.23–1.25
Prevalence of Obesity (scaled)	3.85	3.76–3.95	1.62	1.55–1.69	1.66	1.61–1.72	1.81	1.76–1.86
Region: 1 (as opposed to 4)	1.85	1.75–1.95	0.93	0.83–1.04	1.40	1.30–1.50	1.64	1.55–1.73
Region: 2 (as opposed to 4)	2.19	2.10–2.29	1.44	1.35–1.55	1.39	1.31–1.47	1.43	1.36–1.49
Region: 3 (as opposed to 4)	1.32	1.30–1.33	0.99	0.97–1.01	1.14	1.12–1.16	1.33	1.32–1.35
Region: 5 (as opposed to 4)	1.03	1.03–1.04	0.87	0.85–0.88	1.05	1.03–1.06	1.22	1.20–1.23
Region: 6 (as opposed to 4)	1.12	1.11–1.13	1.13	1.11–1.14	1.02	1.01–1.03	1.04	1.03–1.05
Region: 7 (as opposed to 4)	1.19	1.17–1.21	0.93	0.90–0.96	1.10	1.08–1.12	1.16	1.14–1.18
Region: 8 (as opposed to 4)	2.51	2.45–2.58	0.97	0.92–1.02	1.41	1.36–1.47	1.67	1.62–1.72
Region: 9 (as opposed to 4)	1.88	1.86–1.91	1.28	1.24–1.32	1.25	1.22–1.28	1.49	1.47–1.52
Region: 10 (as opposed to 4)	1.89	1.84–1.95	0.83	0.78–0.88	1.25	1.20–1.30	1.46	1.42–1.51

*Abbreviations*: *Confidence Interval (CI); Coronary Artery Disease (CAD); Chronic Obstructive Pulmonary Disease (COPD); Congestive Heart Failure (CHF); Emergency Department (ED); Fee-For-Service (FFS); Health Maintenance Organization (HMO); Odds Ratio (OR); Preferred Provider Organization (PPO)*

Additional univariate analyses were conducted to characterize the association between a prior year history of anxiety or depression and the presence of subsequent claims for chest imaging, neuroimaging, and imaging within an ED setting. Chest imaging utilization was significantly associated with a history of anxiety (7.2% vs. 5.9%, *P* < .001) and depression (7.4% vs. 5.9%, *P* < .001). Neuroimaging utilization followed the same pattern; patients with a history of anxiety (18.1% vs. 10.3%, *P* < .001) and depression (20.0% vs. 10.0%, *P* < .001) were significantly more likely to have neuroimaging. Lastly, patients with a history of anxiety (32.8% vs. 19.8%, *P* < .001) and depression (33.8% vs. 19.6%, *P* < .001) were significantly more likely to have ED imaging. Adjusted analyses (Table 2) similarly showed that both anxiety and depression were associated with significantly increased odds of chest imaging, neuroimaging, and imaging in an ED setting.

As the majority of the patients were aged 65 years or older, age stratified analyses were conducted so that associations could be examined in the context of patients other age bands. In each age band considered, the adjusted analysis (Table 3) showed that patients with a history of anxiety or depression had significantly increased odds of having imaging in 2018. Depending upon the age band considered, a history of anxiety was associated with between 1.15 to 1.32 increased odds of imaging, and a history of depression was associated with between 1.32 to 1.44 increased odds of imaging.

**Table 3:** Adjusted odds ratios for factors influencing any imaging, by age.

	Age 18–29		Age 30–44		Age 45–64		Age 65+	
	*(n = 24*,*151)*		*(n = 69*,*494)*		*(n = 398*,*295)*		*(n = 1*,*889*,*911)*	
**Variable**	**OR**	**95% CI**	**OR**	**95% CI**	**OR**	**95% CI**	**OR**	**95% CI**
Anxiety	**1.32**	**1.19–1.46**	**1.15**	**1.08–1.23**	**1.22**	**1.18–1.26**	**1.27**	**1.24–1.30**
Depression	**1.32**	**1.18–1.47**	**1.36**	**1.29–1.45**	**1.36**	**1.32–1.39**	**1.44**	**1.42–1.47**
Age	**1.02**	**1.01–1.02**	**1.01**	**1.01–1.02**	**1.01**	**1.01–1.01**	**1.00**	**1.00–1.00**
Rural *(as opposed to Urban)*	**1.01**	**0.92–1.12**	**1.10**	**1.05–1.16**	**1.05**	**1.03–1.07**	**0.97**	**0.96–0.97**
Commercial *(as opposed to Medicare)*	**0.88**	**0.77–1.00**	**0.78**	**0.75–0.81**	**0.94**	**0.93–0.96**	**1.50**	**1.44–1.55**
Plan Type: *HMO (as opposed to FFS)*	**0.57**	**0.22–1.49**	**0.82**	**0.68–0.97**	**0.74**	**0.70–0.78**	**0.71**	**0.69–0.72**
Plan Type: *PPO (as opposed to FFS)*	**0.58**	**0.22–1.51**	**0.91**	**0.76–1.09**	**0.98**	**0.93–1.02**	**1.13**	**1.11–1.15**
Female	**1.50**	**1.42–1.59**	**1.80**	**1.74–1.86**	**1.52**	**1.50–1.54**	**1.37**	**1.36–1.38**
Below $40k Income	**1.02**	**0.93–1.11**	**0.95**	**0.91–0.99**	**0.95**	**0.93–0.97**	**0.96**	**0.95–0.97**
2017 Cardiology Claims	**1.37**	**1.29–1.46**	**1.67**	**1.61–1.73**	**2.27**	**2.23–2.31**	**3.80**	**3.76–3.83**
2017 Imaging Claims	**2.40**	**2.27–2.55**	**2.72**	**2.63–2.81**	**3.44**	**3.39–3.50**	**3.17**	**3.15–3.19**
2017 Oncology Claims	**2.24**	**1.42–3.53**	**2.52**	**1.99–3.18**	**2.32**	**2.14–2.51**	**1.79**	**1.74–1.84**
2017 CAD	**2.13**	**1.33–3.42**	**1.18**	**1.06–1.31**	**1.17**	**1.14–1.20**	**1.21**	**1.20–1.22**
2017 CHF	**1.37**	**0.74–2.55**	**1.40**	**1.22–1.62**	**1.20**	**1.16–1.24**	**1.16**	**1.14–1.17**
2017 COPD	**1.29**	**0.90–1.87**	**1.23**	**1.11–1.36**	**1.25**	**1.22–1.28**	**1.33**	**1.32–1.35**
2017 Diabetes	**1.00**	**0.87–1.16**	**1.08**	**1.02–1.13**	**1.07**	**1.05–1.09**	**1.01**	**1.00–1.01**
Prevalence of Obesity in State (Scaled)	**1.25**	**0.95–1.64**	**1.75**	**1.51–2.03**	**2.90**	**2.73–3.08**	**4.22**	**4.11–4.34**
Region: 1 (as opposed to 4)	**0.92**	**0.35–2.41**	**1.27**	**0.81–2.01**	**1.55**	**1.33–1.8**	**1.95**	**1.84–2.07**
Region: 2 (as opposed to 4)	**1.00**	**0.50–1.98**	**1.34**	**0.97–1.84**	**1.89**	**1.70–2.10**	**2.29**	**2.19–2.40**
Region: 3 (as opposed to 4)	**0.97**	**0.65–1.46**	**1.25**	**1.12–1.40**	**1.31**	**1.27–1.35**	**1.31**	**1.29–1.33**
Region: 5 (as opposed to 4)	**0.92**	**0.85–1.00**	**0.94**	**0.89–0.99**	**0.98**	**0.96–1.00**	**1.05**	**1.04–1.06**
Region: 6 (as opposed to 4)	**0.92**	**0.85–0.99**	**1.00**	**0.96–1.05**	**1.06**	**1.04–1.09**	**1.13**	**1.12–1.14**
Region: 7 (as opposed to 4)	**0.88**	**0.77–1.01**	**0.87**	**0.80–0.94**	**0.99**	**0.95–1.03**	**1.29**	**1.27–1.32**
Region: 8 (as opposed to 4)	**1.03**	**0.78–1.35**	**1.35**	**1.16–1.57**	**2.12**	**1.98–2.27**	**2.66**	**2.58–2.74**
Region: 9 (as opposed to 4)	**0.99**	**0.82–1.19**	**1.10**	**0.99–1.22**	**1.48**	**1.43–1.54**	**2.04**	**2.01–2.08**
Region: 10 (as opposed to 4)	**0.79**	**0.36–1.73**	**1.18**	**0.91–1.52**	**1.74**	**1.60–1.90**	**1.96**	**1.90–2.02**

*Abbreviations*: *Confidence Interval (CI); Coronary Artery Disease (CAD); Chronic Obstructive Pulmonary Disease (COPD); Congestive Heart Failure (CHF); Emergency Department (ED); Fee-For-Service (FFS); Health Maintenance Organization (HMO); Odds Ratio (OR); Preferred Provider Organization (PPO)*

For each of the health statuses examined (history of anxiety, no history of anxiety, history of depression, no history of depression, all), there was a significant, positive association between age and likelihood that an individual would receive imaging, as well as the number of images received, as is shown in Table 4. Among the overall population, a year of age was associated with 1.0063 odds (CI: 1.0060–1.0065) of any imaging. The positive association between a year of age and receipt of any imaging was stronger for patients with a history of anxiety (OR: 1.0168; CI: 1.0157–1.0179) or depression (OR: 1.0127; CI: 1.0117–1.0137), and weaker for patients without a history of anxiety (OR: 1.0067; CI: 1.0065–1.0070) or depression (OR: 1.0076; CI: 1.0074–1.0078).

**Table 4:** Percentage of people receiving imaging and quantity*, by age and health status.

	Age 18–29		Age 30–44		Age 45–64		Age 65+		All Ages		*P*-Value	
	*(n = 24*,*151)*		*(n = 69*,*494)*		*(n = 398*,*295)*		*(n = 1*,*889*,*911)*		*(N = 2*,*381*,*851)*			
**Status**	***%***	*Images*	***%***	*Images*	***%***	*Images*	***%***	*Images*	***%***	*Images*	***%***	*Images*
Anxiety	44.50%	2.13	55.10%	4.32	71.80%	6.72	73.20%	6.35	71.10%	6.23	< .001	< .001
No Anxiety	32.90%	1.28	44.70%	2.42	57.40%	4.00	56.00%	3.77	55.70%	3.74	< .001	< .001
Depression	45.50%	2.36	63.60%	5.52	73.60%	6.87	74.50%	6.54	73.20%	6.54	< .001	< .001
No Depression	32.90%	1.28	43.80%	2.26	56.40%	3.84	55.70%	3.73	55.30%	3.68	< .001	< .001
All	33.80%	1.35	45.70%	2.57	58.30%	4.17	56.40%	3.84	56.20%	3.83	< .001	< .001

*P-Values calculated by testing for a univariate association with age. *Logistic regression used for any imaging; Poisson for # of images*.

Regardless of health status, patients aged 45 to 64 on average received the most images. Likelihood of imaging peaked within the 45 to 64 age band for patients with both a history of anxiety and a history of depression. An adjusted Poisson regression considering the full sample before matching additionally found that the quantity of imaging received was significantly and positively associated with both presence of a history of anxiety (*P*<0.001) and a history of depression (*P*<0.001).

### Findings from the matched sample not consider race

Using CEM, a matched sample consisting of 1,888,159 patients was created. As is shown in Table 1, after weighting the data, there was not a significant association between whether patients had a history of mental illness (anxiety or depression) and their covariate values. Multivariate logistic regression (Table 5) found that a history of anxiety was significantly and positively associated with any imaging (1.18; 95% CI: 1.16–1.20), chest imaging (1.07; 95% CI: 1.04–1.10), neuroimaging (1.39; 95% CI: 1.36–1.42), and ED imaging (1.37; 95% CI: 1.34–1.39). Likewise, a history of depression was significantly and positively associated with any imaging (1.33; 95% CI: 1.32–1.35), chest imaging (1.08; 95% CI: 1.06–1.11), neuroimaging (1.65; 95% CI: 1.63–1.68), and ED imaging (1.41; 95% CI: 1.40–1.43). Thus, the findings were directionally the same after matching.

**Table 5:** Adjusted odds ratios for factors influencing use of any imaging (sample after matching).

	Any Imaging		Chest Imaging		Neuroimaging Imaging		ED Imaging	
Variable	OR	95% CI	OR	95% CI	OR	95% CI	OR	95% CI
Anxiety	1.18	1.16–1.20	1.07	1.04–1.10	1.39	1.36–1.42	1.37	1.34–1.39
Depression	1.33	1.32–1.35	1.08	1.06–1.11	1.65	1.63–1.68	1.41	1.40–1.43
Age	1.00	1.00–1.00	1.01	1.00–1.01	1.00	1.00–1.00	0.99	0.99–0.99
Rural *(as opposed to Urban)*	0.98	0.97–0.99	0.98	0.97–1.00	0.92	0.91–0.93	1.02	1.01–1.03
Commercial *(as opposed to Medicare)*	0.73	0.72–0.74	0.59	0.57–0.61	0.51	0.50–0.52	0.49	0.49–0.50
Plan Type: HMO *(as opposed to FFS)*	0.83	0.82–0.85	0.94	0.91–0.98	0.89	0.86–0.91	0.99	0.97–1.01
Plan Type: PPO *(as opposed to FFS)*	1.06	1.04–1.08	1.12	1.08–1.16	0.98	0.95–1.00	1.00	0.98–1.02
Female	1.48	1.47–1.49	0.95	0.93–0.96	1.19	1.18–1.20	1.16	1.15–1.17
Below $40k Income	0.98	0.97–0.98	1.01	0.99–1.02	1.01	1.00–1.02	1.08	1.08–1.09
2017 Cardiology Claims	2.42	2.39–2.44	2.27	2.21–2.33	1.81	1.78–1.85	1.81	1.79–1.84
2017 Imaging Claims	3.00	2.98–3.02	1.71	1.67–1.74	2.01	1.99–2.04	2.09	2.07–2.12
2017 Oncology Claims	1.91	1.84–1.99	0.95	0.91–1.00	1.44	1.40–1.49	1.23	1.20–1.27
2017 CAD	1.27	1.26–1.28	2.52	2.48–2.55	1.31	1.29–1.32	1.39	1.38–1.40
2017 CHF	1.20	1.19–1.22	1.17	1.15–1.19	1.42	1.40–1.44	1.70	1.68–1.72
2017 COPD	1.47	1.46–1.49	1.18	1.16–1.20	1.23	1.22–1.24	1.60	1.59–1.62
2017 Diabetes	1.08	1.07–1.09	1.32	1.31–1.34	1.20	1.18–1.21	1.28	1.27–1.29
Prevalence of Obesity	2.06	2.01–2.12	1.25	1.20–1.31	1.15	1.11–1.19	1.25	1.21–1.28
Region: 1 *(as opposed to 4)*	1.16	1.10–1.23	0.74	0.65–0.83	0.92	0.86–1.00	1.21	1.14–1.28
Region: 2 *(as opposed to 4)*	1.40	1.33–1.46	1.29	1.20–1.39	1.01	0.95–1.07	0.99	0.94–1.04
Region: 3 *(as opposed to 4)*	1.04	1.03–1.06	0.96	0.94–0.98	1.02	1.01–1.04	1.17	1.15–1.18
Region: 5 *(as opposed to 4)*	0.84	0.84–0.85	0.83	0.81–0.84	0.95	0.94–0.97	1.08	1.07–1.09
Region: 6 *(as opposed to 4)*	0.98	0.98–0.99	1.07	1.05–1.09	0.97	0.96–0.98	0.98	0.97–0.99
Region: 7 *(as opposed to 4)*	0.99	0.97–1.00	0.88	0.85–0.90	1.01	0.99–1.04	1.02	1.00–1.04
Region: 8 *(as opposed to 4)*	1.43	1.39–1.47	0.76	0.72–0.81	1.05	1.02–1.10	1.20	1.16–1.23
Region: 9 *(as opposed to 4)*	1.40	1.37–1.44	1.27	1.22–1.32	1.10	1.06–1.13	1.14	1.11–1.16
Region: 10 *(as opposed to 4)*	1.26	1.23–1.30	0.68	0.63–0.73	1.04	1.00–1.08	1.11	1.08–1.15

*Abbreviations*: *Confidence Interval (CI); Coronary Artery Disease (CAD); Chronic Obstructive Pulmonary Disease (COPD); Congestive Heart Failure (CHF); Emergency Department (ED); Fee-For-Service (FFS); Health Maintenance Organization (HMO); Odds Ratio (OR); Preferred Provider Organization (PPO)*

### Findings from the matched sample considering race

A second matched sample of 1,522,986 patients with Medicare Advantage health plans was created to examine the impact of race. As is shown in Table 6, a history of anxiety and a history of depression were both significantly and positively associated with each of the four dependent variables. Relative to White patients, patients that were Asian, Black, Hispanic, classified as Other, or of an unknown race, were significantly less likely to receive any imaging. Patterns were slightly different for the other forms of imaging. Relative to White patients, Black and Native American patients were significantly more likely to receive chest imaging, while Hispanic patients were significantly less likely to receive chest imaging. Black patients were significantly more likely to receive neuroimaging, relative to White patients, while Asian patients, patients classified as Other, and patients of unknown race were significantly less likely. ED imaging followed a similar pattern to neuroimaging, with the addition that relative to White patients, Native American patients were significantly more likely to receive ED imaging.

**Table 6:** Adjusted odds ratios for factors, including race, influencing use of any imaging by patients with Medicare advantage health plans (sample after matching).

	Any Imaging		Chest Imaging		Neuroimaging Imaging		ED Imaging	
Variable	OR	95% CI	OR	95% CI	OR	95% CI	OR	95% CI
Anxiety	1.19	1.17–1.22	1.07	1.04–1.10	1.39	1.36–1.42	1.39	1.37–1.42
Depression	1.33	1.31–1.35	1.08	1.06–1.11	1.67	1.64–1.70	1.43	1.41–1.45
Age	0.99	0.99–0.99	1.00	1.00–1.00	1.00	1.00–1.00	0.99	0.99–0.99
Rural (as opposed to Urban)	0.95	0.94–0.96	0.97	0.95–0.98	0.92	0.90–0.93	1.03	1.02–1.04
Plan Type: HMO (as opposed to FFS)	0.83	0.81–0.85	0.95	0.91–0.99	0.88	0.85–0.90	0.98	0.95–1.00
Plan Type: PPO (as opposed to FFS)	1.09	1.06–1.11	1.16	1.12–1.21	0.96	0.94–0.99	1.02	0.99–1.04
Female	1.46	1.45–1.47	0.95	0.93–0.96	1.18	1.17–1.20	1.15	1.14–1.16
Below $40k Income	0.97	0.96–0.98	1.00	0.98–1.01	1.01	1.00–1.02	1.03	1.03–1.04
2017 Cardiology Claims	2.78	2.75–2.81	2.32	2.24–2.39	1.90	1.86–1.94	1.90	1.87–1.92
2017 Imaging Claims	2.98	2.95–3.00	1.64	1.60–1.67	2.01	1.98–2.04	2.09	2.06–2.11
2017 Oncology Claims	1.86	1.78–1.93	0.99	0.94–1.04	1.45	1.40–1.51	1.27	1.23–1.31
2017 CAD	1.27	1.26–1.29	2.48	2.44–2.51	1.31	1.29–1.32	1.40	1.38–1.41
2017 CHF	1.22	1.20–1.24	1.17	1.15–1.19	1.40	1.38–1.42	1.64	1.62–1.66
2017 COPD	1.46	1.45–1.48	1.17	1.16–1.19	1.24	1.22–1.25	1.63	1.61–1.64
2017 Diabetes	1.07	1.06–1.08	1.30	1.28–1.32	1.20	1.19–1.21	1.27	1.26–1.28
Prevalence of Obesity	2.36	2.29–2.43	1.35	1.29–1.42	1.23	1.19–1.28	1.32	1.28–1.36
Region: 1 *(as opposed to 4)*	1.36	1.28–1.44	0.95	0.84–1.06	1.08	1.00–1.17	1.36	1.28–1.44
Region: 2 *(as opposed to 4)*	1.52	1.45–1.60	1.21	1.11–1.32	1.10	1.03–1.17	1.02	0.96–1.07
Region: 3 *(as opposed to 4)*	1.05	1.04–1.07	0.97	0.95–0.99	1.02	1.00–1.04	1.16	1.15–1.18
Region: 5 *(as opposed to 4)*	0.86	0.85–0.87	0.82	0.81–0.84	0.96	0.95–0.98	1.09	1.08–1.11
Region: 6 *(as opposed to 4)*	1.00	0.99–1.01	1.08	1.06–1.10	0.97	0.96–0.99	0.98	0.97–0.99
Region: 7 *(as opposed to 4)*	1.04	1.02–1.06	0.87	0.83–0.90	1.02	0.99–1.04	1.05	1.03–1.07
Region: 8 *(as opposed to 4)*	1.57	1.52–1.62	0.81	0.76–0.86	1.10	1.06–1.15	1.28	1.24–1.32
Region: 9 *(as opposed to 4)*	1.53	1.49–1.57	1.36	1.30–1.42	1.15	1.11–1.19	1.19	1.16–1.22
Region: 10 *(as opposed to 4)*	1.32	1.27–1.36	0.71	0.66–0.76	1.04	1.00–1.09	1.12	1.08–1.16
Race: Unknown *(as opposed to White)*	0.94	0.89–0.98	0.92	0.83–1.02	0.72	0.66–0.78	0.61	0.57–0.65
Race: Black *(as opposed to White)*	0.96	0.95–0.97	1.08	1.05–1.10	1.04	1.03–1.06	1.25	1.23–1.27
Race: Other *(as opposed to White)*	0.73	0.70–0.77	0.91	0.83–1.00	0.75	0.70–0.80	0.79	0.74–0.83
Race: Asian *(as opposed to White)*	0.69	0.63–0.75	1.00	0.84–1.19	0.67	0.58–0.78	0.54	0.48–0.61
Race: Hispanic *(as opposed to White)*	0.74	0.70–0.77	0.75	0.67–0.84	1.01	0.94–1.09	1.02	0.97–1.08
Race: Native American *(as opposed to White)*	0.94	0.82–1.08	1.58	1.31–1.89	1.12	0.96–1.31	1.29	1.14–1.46

*Abbreviations*: *Confidence Interval (CI); Coronary Artery Disease (CAD); Chronic Obstructive Pulmonary Disease (COPD); Congestive Heart Failure (CHF); Emergency Department (ED); Fee-For-Service (FFS); Health Maintenance Organization (HMO); Odds Ratio (OR); Preferred Provider Organization (PPO)*

## Discussion

The findings of this study suggest that a history of anxiety or depression in the prior calendar year is associated with greater odds of diagnostic imaging utilization. These findings are directionally opposite to those from the literature on the impact of anxiety and depression on screening imaging, which has found that mental illness is associated with decreased screening imaging [4, 5]. These findings were robust, in that they were maintained regardless of whether the raw sample or a matched sample was analyzed, and held for each of the age strata examined.

As an association was found between a history of mental illness and increased likelihood of chest imaging, neuroimaging, and imaging within the ED, it suggests that there may be a variety of factors causing patients to be more likely to use imaging. Prior research has shown that major depression is often comorbid with ischemic heart disease, and that both depression and psychological stress predispose people to cardiovascular disease [20]. Thus, it is possible that the elevated levels of chest imaging experienced by people with a history of mental illness are a byproduct of the linkage between physical and mental illness [21]. Furthermore, depression and anxiety have been found to be independently associated with recurrent chest pain in low to moderate risk ED patients [22]. Bouts of chest pain could lead patients to present in the ED for imaging. Consequently, patients with a history of coronary artery disease, with a history of congestive heart failure, and with a history of cardiology claims were significantly more likely to need all the categories of imaging examined (Tables 2, 5, & 6).

There are several potential explanations for the finding of a positive association between a history of anxiety or depression and the utilization of diagnostic imaging. First, anxiety or depression may increase patients’ subsequent physical illness [21]. The analysis controlled for several physical comorbidities measured in the prior year, but some patients may have developed subsequent comorbidities because of a reduction in physical activity or in the quality of sleep or diet, caused by anxiety or depression. Second, anxiety or depression may increase patients’ perceptions that they have physical illnesses, and thus increase their likelihood of seeking imaging [22, 23]. Screening imaging is different from diagnostic imaging, in that patients receiving screening imaging do not perceive there to be a physical illness, while patients receiving diagnostic imaging seek imaging in response to their belief that they have a physical illness. Thus, patients with a history of anxiety or depression may have differing behaviors towards screening imaging versus diagnostic imaging, due to the difference in their perception of a need for imaging in the two scenarios.

Although the findings of this study did not address whether or not people with a history of anxiety or depression in this population are receiving an amount of diagnostic imaging appropriate for their clinical condition, they do show that people with these histories are accessing imaging to a greater extent than the overall population. This stands in contrast to the literature on screening imaging, where people with a history of mental illness have been shown to access screening imaging less than the overall population. These findings held even after controlling for factors which might explain the need for imaging, such as a history of cancer, diabetes, heart disease, or imaging in the prior year.

### Limitations

The methodology through which histories of anxiety or depression were determined, a review of one year of claims, likely underreports the frequency of these two conditions. To be counted as having these histories, a patient would have to both have one or more encounters with the healthcare system in 2017, and to have had the anxiety or depression documented during at least one encounter. It is possible that people experiencing anxiety or depression might not interact with the healthcare system in a given year, or might not have a clinician document their anxiety or depression in a claim. This imprecision in determining patients’ 2017 histories likely biases the study away from having significant findings, as the “no history” populations may contain a mixture of people with and without histories of anxiety or depression in the prior year. While it is possible that some of the patients that experienced anxiety or depression in 2017 had their symptoms resolve in 2018, and vice-versa, that some patients in the group without a 2017 history of anxiety or depression went on to develop the conditions in 2018, both of types of clinical changes would bias the study away from having significant findings.

There appears to be a greater frequency of missingness of comorbidity information among younger patients and patients with commercial plans. As a result, the sample used in the analysis had a bias towards older patients than would be the case had there been no exclusion criteria. Nonetheless, there the general finding of an association between the use of imaging and a history of anxiety or depression was maintained after creating a matched sample (Tables 5 and 6), and was observed in each of the age strata when the analysis was stratified by age band (Table 3).

As can be seen from the descriptive statistics, the population that was studied was not representative of the American people. Likewise, the subpopulations of patients with histories of anxiety and depression that were included were not representative of the national population of patients with histories of anxiety and depression. Due to the data source used, all patients had a commercial or Medicare Advantage health plan. Any of the patients included in the sample that had a Medicaid health plan had to have been dually-eligible for Medicare Advantage to meet the study’s inclusion criteria. As was shown in Table 3, the majority of the sample was aged 65 or older. However, subsequent analysis (Tables 3 and 4) helped account for the impact that age may have had on the findings. Finally, the health plans included primarily operate in Southern states, and thus there may be a regional bias.

## Conclusions

Within the context of a privately-insured population, histories of anxiety and depression were associated with greater likelihood of diagnostic imaging utilization. This held true for all imaging, chest imaging, neuroimaging, and imaging within an ED setting. This finding stands in contrast to the literature on screening imaging, which shows that people with a history of mental illness receive significantly less screening.

## Supporting information

S1 File (DOCX)
