# Supplementary material for: The association between a history of anxiety or depression and utilization of diagnostic imaging
Source: PLoS One. 2021 Jul 12;16(7):e0254572. doi: 10.1371/journal.pone.0254572 (PMC8274845; doi:10.1371/journal.pone.0254572)
Supplement: S1 File — (DOCX) [file pone.0254572.s001.docx]

# Appendix A: Diagnosis Definitions

| **Group** | **ICD-9 or ICD-10 Code or Family** | **Description** |
| --- | --- | --- |
| anxiety | 300 | anxiety, dissociative and somatoform disorders |
| anxiety | 3000 | anxiety states |
| anxiety | 3001 | dissociative, conversion and factitious disorders |
| anxiety | 3002 | phobic disorders |
| anxiety | 30000 | anxiety state, unspecified |
| anxiety | 30001 | panic disorder without agoraphobia |
| anxiety | 30002 | generalized anxiety disorder |
| anxiety | 30003 | na |
| anxiety | 30004 | na |
| anxiety | 30005 | na |
| anxiety | 30007 | na |
| anxiety | 30008 | na |
| anxiety | 30009 | other anxiety states |
| anxiety | 30010 | hysteria, unspecified |
| anxiety | 30011 | conversion disorder |
| anxiety | 30012 | dissociative amnesia |
| anxiety | 30013 | dissociative fugue |
| anxiety | 30014 | dissociative identity disorder |
| anxiety | 30015 | dissociative disorder or reaction, unspecified |
| anxiety | 30016 | factitious disorder with predominantly psychological signs and symptoms |
| anxiety | 30019 | other and unspecified factitious illness |
| anxiety | 30020 | phobia, unspecified |
| anxiety | 30021 | agoraphobia with panic disorder |
| anxiety | 30022 | agoraphobia without mention of panic attacks |
| anxiety | 30023 | social phobia |
| anxiety | 30028 | na |
| anxiety | 30029 | other isolated or specific phobias |
| anxiety | 300000 | na |
| anxiety | 300001 | na |
| anxiety | 300002 | na |
| anxiety | 300? | na |
| anxiety | 30000V | na |
| anxiety | 3000A | na |
| anxiety | F4000 | agoraphobia, unspecified |
| anxiety | F4001 | agoraphobia with panic disorder |
| anxiety | F4002 | agoraphobia without panic disorder |
| anxiety | F4010 | social phobia, unspecified |
| anxiety | F4011 | social phobia, generalized |
| anxiety | F40210 | arachnophobia |
| anxiety | F40218 | other animal type phobia |
| anxiety | F40220 | fear of thunderstorms |
| anxiety | F40228 | other natural environment type phobia |
| anxiety | F40230 | fear of blood |
| anxiety | F40231 | fear of injections and transfusions |
| anxiety | F40232 | fear of other medical care |
| anxiety | F40233 | fear of injury |
| anxiety | F40240 | claustrophobia |
| anxiety | F40241 | acrophobia |
| anxiety | F40242 | fear of bridges |
| anxiety | F40243 | fear of flying |
| anxiety | F40248 | other situational type phobia |
| anxiety | F40290 | androphobia |
| anxiety | F40291 | gynephobia |
| anxiety | F40298 | other specified phobia |
| anxiety | F408 | other phobic anxiety disorders |
| anxiety | F409 | phobic anxiety disorder, unspecified |
| anxiety | F410 | panic disorder [episodic paroxysmal anxiety] without agoraphobia |
| anxiety | F411 | generalized anxiety disorder |
| anxiety | F413 | other mixed anxiety disorders |
| anxiety | F418 | other specified anxiety disorders |
| anxiety | F419 | anxiety disorder, unspecified |
| chronic obstructive pulmonary disease | 491 | chronic bronchitis |
| chronic obstructive pulmonary disease | 492 | emphysema |
| chronic obstructive pulmonary disease | 496 | chronic airway obstruction, not elsewhere classified |
| chronic obstructive pulmonary disease | 4910 | simple chronic bronchitis |
| chronic obstructive pulmonary disease | 4911 | mucopurulent chronic bronchitis |
| chronic obstructive pulmonary disease | 4912 | obstructive chronic bronchitis |
| chronic obstructive pulmonary disease | 4913 | na |
| chronic obstructive pulmonary disease | 4918 | other chronic bronchitis |
| chronic obstructive pulmonary disease | 4919 | unspecified chronic bronchitis |
| chronic obstructive pulmonary disease | 4920 | emphysematous bleb |
| chronic obstructive pulmonary disease | 4921 | na |
| chronic obstructive pulmonary disease | 4922 | na |
| chronic obstructive pulmonary disease | 4923 | na |
| chronic obstructive pulmonary disease | 4925 | na |
| chronic obstructive pulmonary disease | 4926 | na |
| chronic obstructive pulmonary disease | 4928 | other emphysema |
| chronic obstructive pulmonary disease | 4929 | na |
| chronic obstructive pulmonary disease | 4960 | na |
| chronic obstructive pulmonary disease | 4961 | na |
| chronic obstructive pulmonary disease | 4962 | na |
| chronic obstructive pulmonary disease | 4963 | na |
| chronic obstructive pulmonary disease | 4968 | na |
| chronic obstructive pulmonary disease | 4969 | na |
| chronic obstructive pulmonary disease | 5181 | interstitial emphysema |
| chronic obstructive pulmonary disease | 5182 | compensatory emphysema |
| chronic obstructive pulmonary disease | 49100 | na |
| chronic obstructive pulmonary disease | 49110 | na |
| chronic obstructive pulmonary disease | 49112 | na |
| chronic obstructive pulmonary disease | 49120 | obstructive chronic bronchitis, without exacerbation |
| chronic obstructive pulmonary disease | 49121 | obstructive chronic bronchitis, with (acute) exacerbation |
| chronic obstructive pulmonary disease | 49122 | obstructive chronic bronchitis with acute bronchitis |
| chronic obstructive pulmonary disease | 49123 | na |
| chronic obstructive pulmonary disease | 49126 | na |
| chronic obstructive pulmonary disease | 49141 | na |
| chronic obstructive pulmonary disease | 49181 | na |
| chronic obstructive pulmonary disease | 49190 | na |
| chronic obstructive pulmonary disease | 49191 | na |
| chronic obstructive pulmonary disease | 49221 | na |
| chronic obstructive pulmonary disease | 49280 | na |
| chronic obstructive pulmonary disease | 49286 | na |
| chronic obstructive pulmonary disease | 49290 | na |
| chronic obstructive pulmonary disease | 49291 | na |
| chronic obstructive pulmonary disease | 49292 | na |
| chronic obstructive pulmonary disease | 49293 | na |
| chronic obstructive pulmonary disease | 49600 | na |
| chronic obstructive pulmonary disease | 49620 | na |
| chronic obstructive pulmonary disease | 49638 | na |
| chronic obstructive pulmonary disease | 49639 | na |
| chronic obstructive pulmonary disease | 49665 | na |
| chronic obstructive pulmonary disease | 49672 | na |
| chronic obstructive pulmonary disease | 49690 | na |
| chronic obstructive pulmonary disease | 49692 | na |
| chronic obstructive pulmonary disease | 496300 | na |
| chronic obstructive pulmonary disease | 496C | na |
| chronic obstructive pulmonary disease | 496W | na |
| chronic obstructive pulmonary disease | J410 | simple chronic bronchitis |
| chronic obstructive pulmonary disease | J411 | mucopurulent chronic bronchitis |
| chronic obstructive pulmonary disease | J418 | mixed simple and mucopurulent chronic bronchitis |
| chronic obstructive pulmonary disease | J42 | unspecified chronic bronchitis |
| chronic obstructive pulmonary disease | J430 | unilateral pulmonary emphysema [macleod's syndrome] |
| chronic obstructive pulmonary disease | J431 | panlobular emphysema |
| chronic obstructive pulmonary disease | J432 | centrilobular emphysema |
| chronic obstructive pulmonary disease | J438 | other emphysema |
| chronic obstructive pulmonary disease | J439 | emphysema, unspecified |
| chronic obstructive pulmonary disease | J440 | chronic obstructive pulmonary disease with acute lower respiratory infection |
| chronic obstructive pulmonary disease | J441 | chronic obstructive pulmonary disease with (acute) exacerbation |
| chronic obstructive pulmonary disease | J449 | chronic obstructive pulmonary disease, unspecified |
| chronic obstructive pulmonary disease | J982 | interstitial emphysema |
| chronic obstructive pulmonary disease | J983 | compensatory emphysema |
| congestive heart failure | 428 | heart failure |
| congestive heart failure | 4150 | acute cor pulmonale |
| congestive heart failure | 4280 | congestive heart failure, unspecified |
| congestive heart failure | 4281 | left heart failure |
| congestive heart failure | 4282 | systolic heart failure |
| congestive heart failure | 4283 | diastolic heart failure |
| congestive heart failure | 4284 | combined systolic and diastolic heart failure |
| congestive heart failure | 4285 | na |
| congestive heart failure | 4286 | na |
| congestive heart failure | 4287 | na |
| congestive heart failure | 4288 | na |
| congestive heart failure | 4289 | unspecified heart failure |
| congestive heart failure | 39891 | rheumatic heart failure (congestive) |
| congestive heart failure | 40201 | malignant hypertensive heart disease with heart failure |
| congestive heart failure | 40211 | benign hypertensive heart disease with heart failure |
| congestive heart failure | 40291 | unspecified hypertensive heart disease with heart failure |
| congestive heart failure | 40401 | hypertensive heart and kidney disease, malignant, with heart failure |
| congestive heart failure | 40411 | hypertensive heart and kidney disease, benign, with heart failure |
| congestive heart failure | 40491 | hypertensive heart and kidney disease, unspecified, with heart failure |
| congestive heart failure | 42800 | na |
| congestive heart failure | 42801 | na |
| congestive heart failure | 42802 | na |
| congestive heart failure | 42804 | na |
| congestive heart failure | 42809 | na |
| congestive heart failure | 42810 | na |
| congestive heart failure | 42820 | unspecified systolic heart failure |
| congestive heart failure | 42821 | acute systolic heart failure |
| congestive heart failure | 42822 | chronic systolic heart failure |
| congestive heart failure | 42823 | acute on chronic systolic heart failure |
| congestive heart failure | 42830 | unspecified diastolic heart failure |
| congestive heart failure | 42831 | acute diastolic heart failure |
| congestive heart failure | 42832 | chronic diastolic heart failure |
| congestive heart failure | 42833 | acute on chronic diastolic heart failure |
| congestive heart failure | 42840 | unspecified combined systolic and diastolic heart failure |
| congestive heart failure | 42841 | acute combined systolic and diastolic heart failure |
| congestive heart failure | 42842 | chronic combined systolic and diastolic heart failure |
| congestive heart failure | 42843 | acute on chronic combined systolic and diastolic heart failure |
| congestive heart failure | 42885 | na |
| congestive heart failure | 42898 | na |
| congestive heart failure | 428C | na |
| congestive heart failure | 428D | na |
| congestive heart failure | 428O | na |
| congestive heart failure | I0981 | rheumatic heart failure |
| congestive heart failure | I110 | hypertensive heart disease with heart failure |
| congestive heart failure | I130 | hypertensive heart and chronic kidney disease with heart failure and stage 1 through stage 4 chronic kidney disease, or unspecified chronic kidney disease |
| congestive heart failure | I501 | left ventricular failure |
| congestive heart failure | I5020 | unspecified systolic (congestive) heart failure |
| congestive heart failure | I5021 | acute systolic (congestive) heart failure |
| congestive heart failure | I5022 | chronic systolic (congestive) heart failure |
| congestive heart failure | I5023 | acute on chronic systolic (congestive) heart failure |
| congestive heart failure | I5030 | unspecified diastolic (congestive) heart failure |
| congestive heart failure | I5031 | acute diastolic (congestive) heart failure |
| congestive heart failure | I5032 | chronic diastolic (congestive) heart failure |
| congestive heart failure | I5033 | acute on chronic diastolic (congestive) heart failure |
| congestive heart failure | I5040 | unspecified combined systolic (congestive) and diastolic (congestive) heart failure |
| congestive heart failure | I5041 | acute combined systolic (congestive) and diastolic (congestive) heart failure |
| congestive heart failure | I5042 | chronic combined systolic (congestive) and diastolic (congestive) heart failure |
| congestive heart failure | I5043 | acute on chronic combined systolic (congestive) and diastolic (congestive) heart failure |
| congestive heart failure | I50810 | right heart failure, unspecified |
| congestive heart failure | I50811 | acute right heart failure |
| congestive heart failure | I50812 | chronic right heart failure |
| congestive heart failure | I50813 | acute on chronic right heart failure |
| congestive heart failure | I50814 | right heart failure due to left heart failure |
| congestive heart failure | I5082 | biventricular heart failure |
| congestive heart failure | I5083 | high output heart failure |
| congestive heart failure | I5084 | end stage heart failure |
| congestive heart failure | I5089 | other heart failure |
| congestive heart failure | I509 | heart failure, unspecified |
| coronary artery disease | 41 | na |
| coronary artery disease | 410 | acute myocardial infarction |
| coronary artery disease | 411 | other acute and subacute forms of ischemic heart disease |
| coronary artery disease | 412 | old myocardial infarction |
| coronary artery disease | 413 | angina pectoris |
| coronary artery disease | 414 | other forms of chronic ischemic heart disease |
| coronary artery disease | 428 | heart failure |
| coronary artery disease | 491 | chronic bronchitis |
| coronary artery disease | 492 | emphysema |
| coronary artery disease | 496 | chronic airway obstruction, not elsewhere classified |
| coronary artery disease | 4100 | acute myocardial infarction of anterolateral wall |
| coronary artery disease | 4101 | acute myocardial infarction of other anterior wall |
| coronary artery disease | 4102 | acute myocardial infarction of inferolateral wall |
| coronary artery disease | 4103 | acute myocardial infarction of inferoposterior wall |
| coronary artery disease | 4104 | acute myocardial infarction of other inferior wall |
| coronary artery disease | 4105 | acute myocardial infarction of other lateral wall |
| coronary artery disease | 4106 | acute myocardial infarction, true posterior wall infarction |
| coronary artery disease | 4107 | acute myocardial infarction, subendocardial infarction |
| coronary artery disease | 4108 | acute myocardial infarction of other specified sites |
| coronary artery disease | 4109 | acute myocardial infarction, unspecified site |
| coronary artery disease | 4110 | postmyocardial infarction syndrome |
| coronary artery disease | 4111 | intermediate coronary syndrome |
| coronary artery disease | 4112 | na |
| coronary artery disease | 4114 | na |
| coronary artery disease | 4116 | na |
| coronary artery disease | 4118 | other acute and subacute forms of ischemic heart disease |
| coronary artery disease | 4119 | na |
| coronary artery disease | 4120 | na |
| coronary artery disease | 4123 | na |
| coronary artery disease | 4128 | na |
| coronary artery disease | 4129 | na |
| coronary artery disease | 4130 | angina decubitus |
| coronary artery disease | 4131 | prinzmetal angina |
| coronary artery disease | 4132 | na |
| coronary artery disease | 4133 | na |
| coronary artery disease | 4134 | na |
| coronary artery disease | 4135 | na |
| coronary artery disease | 4138 | na |
| coronary artery disease | 4139 | other and unspecified angina pectoris |
| coronary artery disease | 4140 | coronary atherosclerosis |
| coronary artery disease | 4141 | aneurysm and dissection of heart |
| coronary artery disease | 4142 | chronic total occlusion of coronary artery |
| coronary artery disease | 4143 | coronary atherosclerosis due to lipid rich plaque |
| coronary artery disease | 4144 | coronary atherosclerosis due to calcified coronary lesion |
| coronary artery disease | 4145 | na |
| coronary artery disease | 4146 | na |
| coronary artery disease | 4148 | other specified forms of chronic ischemic heart disease |
| coronary artery disease | 4149 | unspecified chronic ischemic heart disease |
| coronary artery disease | 4150 | acute cor pulmonale |
| coronary artery disease | 4280 | congestive heart failure, unspecified |
| coronary artery disease | 4281 | left heart failure |
| coronary artery disease | 4282 | systolic heart failure |
| coronary artery disease | 4283 | diastolic heart failure |
| coronary artery disease | 4284 | combined systolic and diastolic heart failure |
| coronary artery disease | 4285 | na |
| coronary artery disease | 4286 | na |
| coronary artery disease | 4287 | na |
| coronary artery disease | 4288 | na |
| coronary artery disease | 4289 | unspecified heart failure |
| coronary artery disease | 4295 | rupture of chordae tendineae |
| coronary artery disease | 4296 | rupture of papillary muscle |
| coronary artery disease | 4910 | simple chronic bronchitis |
| coronary artery disease | 4911 | mucopurulent chronic bronchitis |
| coronary artery disease | 4912 | obstructive chronic bronchitis |
| coronary artery disease | 4913 | na |
| coronary artery disease | 4918 | other chronic bronchitis |
| coronary artery disease | 4919 | unspecified chronic bronchitis |
| coronary artery disease | 4920 | emphysematous bleb |
| coronary artery disease | 4921 | na |
| coronary artery disease | 4922 | na |
| coronary artery disease | 4923 | na |
| coronary artery disease | 4925 | na |
| coronary artery disease | 4926 | na |
| coronary artery disease | 4928 | other emphysema |
| coronary artery disease | 4929 | na |
| coronary artery disease | 4960 | na |
| coronary artery disease | 4961 | na |
| coronary artery disease | 4962 | na |
| coronary artery disease | 4963 | na |
| coronary artery disease | 4968 | na |
| coronary artery disease | 4969 | na |
| coronary artery disease | 5181 | interstitial emphysema |
| coronary artery disease | 5182 | compensatory emphysema |
| coronary artery disease | 39891 | rheumatic heart failure (congestive) |
| coronary artery disease | 40201 | malignant hypertensive heart disease with heart failure |
| coronary artery disease | 40211 | benign hypertensive heart disease with heart failure |
| coronary artery disease | 40291 | unspecified hypertensive heart disease with heart failure |
| coronary artery disease | 40401 | hypertensive heart and kidney disease, malignant, with heart failure |
| coronary artery disease | 40411 | hypertensive heart and kidney disease, benign, with heart failure |
| coronary artery disease | 40491 | hypertensive heart and kidney disease, unspecified, with heart failure |
| coronary artery disease | 41000 | acute myocardial infarction of anterolateral wall, episode of care unspecified |
| coronary artery disease | 41001 | acute myocardial infarction of anterolateral wall, initial episode of care |
| coronary artery disease | 41002 | acute myocardial infarction of anterolateral wall, subsequent episode of care |
| coronary artery disease | 41010 | acute myocardial infarction of other anterior wall, episode of care unspecified |
| coronary artery disease | 41011 | acute myocardial infarction of other anterior wall, initial episode of care |
| coronary artery disease | 41012 | acute myocardial infarction of other anterior wall, subsequent episode of care |
| coronary artery disease | 41017 | na |
| coronary artery disease | 41019 | na |
| coronary artery disease | 41020 | acute myocardial infarction of inferolateral wall, episode of care unspecified |
| coronary artery disease | 41021 | acute myocardial infarction of inferolateral wall, initial episode of care |
| coronary artery disease | 41022 | acute myocardial infarction of inferolateral wall, subsequent episode of care |
| coronary artery disease | 41030 | acute myocardial infarction of inferoposterior wall, episode of care unspecified |
| coronary artery disease | 41031 | acute myocardial infarction of inferoposterior wall, initial episode of care |
| coronary artery disease | 41032 | acute myocardial infarction of inferoposterior wall, subsequent episode of care |
| coronary artery disease | 41040 | acute myocardial infarction of other inferior wall, episode of care unspecified |
| coronary artery disease | 41041 | acute myocardial infarction of other inferior wall, initial episode of care |
| coronary artery disease | 41042 | acute myocardial infarction of other inferior wall, subsequent episode of care |
| coronary artery disease | 41050 | acute myocardial infarction of other lateral wall, episode of care unspecified |
| coronary artery disease | 41051 | acute myocardial infarction of other lateral wall, initial episode of care |
| coronary artery disease | 41052 | acute myocardial infarction of other lateral wall, subsequent episode of care |
| coronary artery disease | 41060 | acute myocardial infarction, true posterior wall infarction, episode of care unspecified |
| coronary artery disease | 41061 | acute myocardial infarction, true posterior wall infarction, initial episode of care |
| coronary artery disease | 41062 | acute myocardial infarction, true posterior wall infarction, subsequent episode of care |
| coronary artery disease | 41070 | acute myocardial infarction, subendocardial infarction, episode of care unspecified |
| coronary artery disease | 41071 | acute myocardial infarction, subendocardial infarction, initial episode of care |
| coronary artery disease | 41072 | acute myocardial infarction, subendocardial infarction, subsequent episode of care |
| coronary artery disease | 41080 | acute myocardial infarction of other specified sites, episode of care unspecified |
| coronary artery disease | 41081 | acute myocardial infarction of other specified sites, initial episode of care |
| coronary artery disease | 41082 | acute myocardial infarction of other specified sites, subsequent episode of care |
| coronary artery disease | 41085 | na |
| coronary artery disease | 41089 | na |
| coronary artery disease | 41090 | acute myocardial infarction, unspecified site, episode of care unspecified |
| coronary artery disease | 41091 | acute myocardial infarction, unspecified site, initial episode of care |
| coronary artery disease | 41092 | acute myocardial infarction, unspecified site, subsequent episode of care |
| coronary artery disease | 41094 | na |
| coronary artery disease | 41099 | na |
| coronary artery disease | 41100 | na |
| coronary artery disease | 41101 | na |
| coronary artery disease | 41110 | na |
| coronary artery disease | 41111 | na |
| coronary artery disease | 41117 | na |
| coronary artery disease | 41121 | na |
| coronary artery disease | 41169 | na |
| coronary artery disease | 41171 | na |
| coronary artery disease | 41172 | na |
| coronary artery disease | 41181 | acute coronary occlusion without myocardial infarction |
| coronary artery disease | 41188 | na |
| coronary artery disease | 41189 | other acute and subacute form of ischemic heart disease |
| coronary artery disease | 41271 | na |
| coronary artery disease | 41272 | na |
| coronary artery disease | 41309 | na |
| coronary artery disease | 41310 | na |
| coronary artery disease | 41311 | na |
| coronary artery disease | 41319 | na |
| coronary artery disease | 41353 | na |
| coronary artery disease | 41390 | na |
| coronary artery disease | 41391 | na |
| coronary artery disease | 41399 | na |
| coronary artery disease | 41400 | coronary atherosclerosis of unspecified type of vessel, native or graft |
| coronary artery disease | 41401 | coronary atherosclerosis of native coronary artery |
| coronary artery disease | 41402 | coronary atherosclerosis of autologous vein bypass graft |
| coronary artery disease | 41403 | coronary atherosclerosis of nonautologous biological bypass graft |
| coronary artery disease | 41404 | coronary atherosclerosis of artery bypass graft |
| coronary artery disease | 41405 | coronary atherosclerosis of unspecified type of bypass graft |
| coronary artery disease | 41406 | coronary atherosclerosis, of native coronary artery of transplanted heart |
| coronary artery disease | 41407 | coronary atherosclerosis, of bypass graft (artery) (vein) of transplanted heart |
| coronary artery disease | 41408 | na |
| coronary artery disease | 41409 | na |
| coronary artery disease | 41410 | aneurysm of heart |
| coronary artery disease | 41411 | aneurysm of coronary vessels |
| coronary artery disease | 41412 | dissection of coronary artery |
| coronary artery disease | 41419 | other aneurysm of heart |
| coronary artery disease | 41461 | na |
| coronary artery disease | 41480 | na |
| coronary artery disease | 41481 | na |
| coronary artery disease | 41482 | na |
| coronary artery disease | 41488 | na |
| coronary artery disease | 41489 | na |
| coronary artery disease | 41490 | na |
| coronary artery disease | 41492 | na |
| coronary artery disease | 41494 | na |
| coronary artery disease | 41497 | na |
| coronary artery disease | 41499 | na |
| coronary artery disease | 42800 | na |
| coronary artery disease | 42801 | na |
| coronary artery disease | 42802 | na |
| coronary artery disease | 42804 | na |
| coronary artery disease | 42809 | na |
| coronary artery disease | 42810 | na |
| coronary artery disease | 42820 | unspecified systolic heart failure |
| coronary artery disease | 42821 | acute systolic heart failure |
| coronary artery disease | 42822 | chronic systolic heart failure |
| coronary artery disease | 42823 | acute on chronic systolic heart failure |
| coronary artery disease | 42830 | unspecified diastolic heart failure |
| coronary artery disease | 42831 | acute diastolic heart failure |
| coronary artery disease | 42832 | chronic diastolic heart failure |
| coronary artery disease | 42833 | acute on chronic diastolic heart failure |
| coronary artery disease | 42840 | unspecified combined systolic and diastolic heart failure |
| coronary artery disease | 42841 | acute combined systolic and diastolic heart failure |
| coronary artery disease | 42842 | chronic combined systolic and diastolic heart failure |
| coronary artery disease | 42843 | acute on chronic combined systolic and diastolic heart failure |
| coronary artery disease | 42885 | na |
| coronary artery disease | 42898 | na |
| coronary artery disease | 42962 | na |
| coronary artery disease | 42963 | na |
| coronary artery disease | 49100 | na |
| coronary artery disease | 49110 | na |
| coronary artery disease | 49112 | na |
| coronary artery disease | 49120 | obstructive chronic bronchitis, without exacerbation |
| coronary artery disease | 49121 | obstructive chronic bronchitis, with (acute) exacerbation |
| coronary artery disease | 49122 | obstructive chronic bronchitis with acute bronchitis |
| coronary artery disease | 49123 | na |
| coronary artery disease | 49126 | na |
| coronary artery disease | 49141 | na |
| coronary artery disease | 49181 | na |
| coronary artery disease | 49190 | na |
| coronary artery disease | 49191 | na |
| coronary artery disease | 49221 | na |
| coronary artery disease | 49280 | na |
| coronary artery disease | 49286 | na |
| coronary artery disease | 49290 | na |
| coronary artery disease | 49291 | na |
| coronary artery disease | 49292 | na |
| coronary artery disease | 49293 | na |
| coronary artery disease | 49600 | na |
| coronary artery disease | 49620 | na |
| coronary artery disease | 49638 | na |
| coronary artery disease | 49639 | na |
| coronary artery disease | 49665 | na |
| coronary artery disease | 49672 | na |
| coronary artery disease | 49690 | na |
| coronary artery disease | 49692 | na |
| coronary artery disease | 414010 | na |
| coronary artery disease | 414016 | na |
| coronary artery disease | 496300 | na |
| coronary artery disease | 4139V | na |
| coronary artery disease | 428C | na |
| coronary artery disease | 428D | na |
| coronary artery disease | 428O | na |
| coronary artery disease | 496C | na |
| coronary artery disease | 496W | na |
| coronary artery disease | I0981 | rheumatic heart failure |
| coronary artery disease | I110 | hypertensive heart disease with heart failure |
| coronary artery disease | I130 | hypertensive heart and chronic kidney disease with heart failure and stage 1 through stage 4 chronic kidney disease, or unspecified chronic kidney disease |
| coronary artery disease | I200 | unstable angina |
| coronary artery disease | I201 | angina pectoris with documented spasm |
| coronary artery disease | I208 | other forms of angina pectoris |
| coronary artery disease | I209 | angina pectoris, unspecified |
| coronary artery disease | I2101 | st elevation (stemi) myocardial infarction involving left main coronary artery |
| coronary artery disease | I2102 | st elevation (stemi) myocardial infarction involving left anterior descending coronary artery |
| coronary artery disease | I2109 | st elevation (stemi) myocardial infarction involving other coronary artery of anterior wall |
| coronary artery disease | I2111 | st elevation (stemi) myocardial infarction involving right coronary artery |
| coronary artery disease | I2119 | st elevation (stemi) myocardial infarction involving other coronary artery of inferior wall |
| coronary artery disease | I2121 | st elevation (stemi) myocardial infarction involving left circumflex coronary artery |
| coronary artery disease | I2129 | st elevation (stemi) myocardial infarction involving other sites |
| coronary artery disease | I213 | st elevation (stemi) myocardial infarction of unspecified site |
| coronary artery disease | I214 | non-st elevation (nstemi) myocardial infarction |
| coronary artery disease | I219 | acute myocardial infarction, unspecified |
| coronary artery disease | I21A1 | myocardial infarction type 2 |
| coronary artery disease | I21A9 | other myocardial infarction type |
| coronary artery disease | I220 | subsequent st elevation (stemi) myocardial infarction of anterior wall |
| coronary artery disease | I221 | subsequent st elevation (stemi) myocardial infarction of inferior wall |
| coronary artery disease | I222 | subsequent non-st elevation (nstemi) myocardial infarction |
| coronary artery disease | I228 | subsequent st elevation (stemi) myocardial infarction of other sites |
| coronary artery disease | I229 | subsequent st elevation (stemi) myocardial infarction of unspecified site |
| coronary artery disease | I234 | rupture of chordae tendineae as current complication following acute myocardial infarction |
| coronary artery disease | I235 | rupture of papillary muscle as current complication following acute myocardial infarction |
| coronary artery disease | I240 | acute coronary thrombosis not resulting in myocardial infarction |
| coronary artery disease | I241 | dressler's syndrome |
| coronary artery disease | I248 | other forms of acute ischemic heart disease |
| coronary artery disease | I249 | acute ischemic heart disease, unspecified |
| coronary artery disease | I2510 | atherosclerotic heart disease of native coronary artery without angina pectoris |
| coronary artery disease | I25110 | atherosclerotic heart disease of native coronary artery with unstable angina pectoris |
| coronary artery disease | I25111 | atherosclerotic heart disease of native coronary artery with angina pectoris with documented spasm |
| coronary artery disease | I25118 | atherosclerotic heart disease of native coronary artery with other forms of angina pectoris |
| coronary artery disease | I25119 | atherosclerotic heart disease of native coronary artery with unspecified angina pectoris |
| coronary artery disease | I252 | old myocardial infarction |
| coronary artery disease | I253 | aneurysm of heart |
| coronary artery disease | I2541 | coronary artery aneurysm |
| coronary artery disease | I2542 | coronary artery dissection |
| coronary artery disease | I255 | ischemic cardiomyopathy |
| coronary artery disease | I256 | silent myocardial ischemia |
| coronary artery disease | I25700 | atherosclerosis of coronary artery bypass graft(s), unspecified, with unstable angina pectoris |
| coronary artery disease | I25701 | atherosclerosis of coronary artery bypass graft(s), unspecified, with angina pectoris with documented spasm |
| coronary artery disease | I25708 | atherosclerosis of coronary artery bypass graft(s), unspecified, with other forms of angina pectoris |
| coronary artery disease | I25709 | atherosclerosis of coronary artery bypass graft(s), unspecified, with unspecified angina pectoris |
| coronary artery disease | I25710 | atherosclerosis of autologous vein coronary artery bypass graft(s) with unstable angina pectoris |
| coronary artery disease | I25711 | atherosclerosis of autologous vein coronary artery bypass graft(s) with angina pectoris with documented spasm |
| coronary artery disease | I25718 | atherosclerosis of autologous vein coronary artery bypass graft(s) with other forms of angina pectoris |
| coronary artery disease | I25719 | atherosclerosis of autologous vein coronary artery bypass graft(s) with unspecified angina pectoris |
| coronary artery disease | I25720 | atherosclerosis of autologous artery coronary artery bypass graft(s) with unstable angina pectoris |
| coronary artery disease | I25721 | atherosclerosis of autologous artery coronary artery bypass graft(s) with angina pectoris with documented spasm |
| coronary artery disease | I25728 | atherosclerosis of autologous artery coronary artery bypass graft(s) with other forms of angina pectoris |
| coronary artery disease | I25729 | atherosclerosis of autologous artery coronary artery bypass graft(s) with unspecified angina pectoris |
| coronary artery disease | I25730 | atherosclerosis of nonautologous biological coronary artery bypass graft(s) with unstable angina pectoris |
| coronary artery disease | I25731 | atherosclerosis of nonautologous biological coronary artery bypass graft(s) with angina pectoris with documented spasm |
| coronary artery disease | I25738 | atherosclerosis of nonautologous biological coronary artery bypass graft(s) with other forms of angina pectoris |
| coronary artery disease | I25739 | atherosclerosis of nonautologous biological coronary artery bypass graft(s) with unspecified angina pectoris |
| coronary artery disease | I25750 | atherosclerosis of native coronary artery of transplanted heart with unstable angina |
| coronary artery disease | I25751 | atherosclerosis of native coronary artery of transplanted heart with angina pectoris with documented spasm |
| coronary artery disease | I25758 | atherosclerosis of native coronary artery of transplanted heart with other forms of angina pectoris |
| coronary artery disease | I25759 | atherosclerosis of native coronary artery of transplanted heart with unspecified angina pectoris |
| coronary artery disease | I25760 | atherosclerosis of bypass graft of coronary artery of transplanted heart with unstable angina |
| coronary artery disease | I25761 | atherosclerosis of bypass graft of coronary artery of transplanted heart with angina pectoris with documented spasm |
| coronary artery disease | I25768 | atherosclerosis of bypass graft of coronary artery of transplanted heart with other forms of angina pectoris |
| coronary artery disease | I25769 | atherosclerosis of bypass graft of coronary artery of transplanted heart with unspecified angina pectoris |
| coronary artery disease | I25790 | atherosclerosis of other coronary artery bypass graft(s) with unstable angina pectoris |
| coronary artery disease | I25791 | atherosclerosis of other coronary artery bypass graft(s) with angina pectoris with documented spasm |
| coronary artery disease | I25798 | atherosclerosis of other coronary artery bypass graft(s) with other forms of angina pectoris |
| coronary artery disease | I25799 | atherosclerosis of other coronary artery bypass graft(s) with unspecified angina pectoris |
| coronary artery disease | I25810 | atherosclerosis of coronary artery bypass graft(s) without angina pectoris |
| coronary artery disease | I25811 | atherosclerosis of native coronary artery of transplanted heart without angina pectoris |
| coronary artery disease | I25812 | atherosclerosis of bypass graft of coronary artery of transplanted heart without angina pectoris |
| coronary artery disease | I2582 | chronic total occlusion of coronary artery |
| coronary artery disease | I2583 | coronary atherosclerosis due to lipid rich plaque |
| coronary artery disease | I2584 | coronary atherosclerosis due to calcified coronary lesion |
| coronary artery disease | I2589 | other forms of chronic ischemic heart disease |
| coronary artery disease | I259 | chronic ischemic heart disease, unspecified |
| coronary artery disease | I501 | left ventricular failure |
| coronary artery disease | I5020 | unspecified systolic (congestive) heart failure |
| coronary artery disease | I5021 | acute systolic (congestive) heart failure |
| coronary artery disease | I5022 | chronic systolic (congestive) heart failure |
| coronary artery disease | I5023 | acute on chronic systolic (congestive) heart failure |
| coronary artery disease | I5030 | unspecified diastolic (congestive) heart failure |
| coronary artery disease | I5031 | acute diastolic (congestive) heart failure |
| coronary artery disease | I5032 | chronic diastolic (congestive) heart failure |
| coronary artery disease | I5033 | acute on chronic diastolic (congestive) heart failure |
| coronary artery disease | I5040 | unspecified combined systolic (congestive) and diastolic (congestive) heart failure |
| coronary artery disease | I5041 | acute combined systolic (congestive) and diastolic (congestive) heart failure |
| coronary artery disease | I5042 | chronic combined systolic (congestive) and diastolic (congestive) heart failure |
| coronary artery disease | I5043 | acute on chronic combined systolic (congestive) and diastolic (congestive) heart failure |
| coronary artery disease | I50810 | right heart failure, unspecified |
| coronary artery disease | I50811 | acute right heart failure |
| coronary artery disease | I50812 | chronic right heart failure |
| coronary artery disease | I50813 | acute on chronic right heart failure |
| coronary artery disease | I50814 | right heart failure due to left heart failure |
| coronary artery disease | I5082 | biventricular heart failure |
| coronary artery disease | I5083 | high output heart failure |
| coronary artery disease | I5084 | end stage heart failure |
| coronary artery disease | I5089 | other heart failure |
| coronary artery disease | I509 | heart failure, unspecified |
| coronary artery disease | I511 | rupture of chordae tendineae, not elsewhere classified |
| coronary artery disease | I512 | rupture of papillary muscle, not elsewhere classified |
| coronary artery disease | J410 | simple chronic bronchitis |
| coronary artery disease | J411 | mucopurulent chronic bronchitis |
| coronary artery disease | J418 | mixed simple and mucopurulent chronic bronchitis |
| coronary artery disease | J42 | unspecified chronic bronchitis |
| coronary artery disease | J430 | unilateral pulmonary emphysema [macleod's syndrome] |
| coronary artery disease | J431 | panlobular emphysema |
| coronary artery disease | J432 | centrilobular emphysema |
| coronary artery disease | J438 | other emphysema |
| coronary artery disease | J439 | emphysema, unspecified |
| coronary artery disease | J440 | chronic obstructive pulmonary disease with acute lower respiratory infection |
| coronary artery disease | J441 | chronic obstructive pulmonary disease with (acute) exacerbation |
| coronary artery disease | J449 | chronic obstructive pulmonary disease, unspecified |
| coronary artery disease | J982 | interstitial emphysema |
| coronary artery disease | J983 | compensatory emphysema |
| coronary artery disease | T82855A | stenosis of coronary artery stent, initial encounter |
| coronary artery disease | T82855D | stenosis of coronary artery stent, subsequent encounter |
| coronary artery disease | T82855S | stenosis of coronary artery stent, sequela |
| coronary artery disease | V4581 | postprocedural aortocoronary bypass status |
| coronary artery disease | V4582 | postprocedural percutaneous transluminal coronary angioplasty status |
| coronary artery disease | Z8342 | family history of familial hypercholesterolemia |
| coronary artery disease | Z951 | presence of aortocoronary bypass graft |
| coronary artery disease | Z955 | presence of coronary angioplasty implant and graft |
| coronary artery disease | Z9861 | coronary angioplasty status |
| depression | 296 | episodic mood disorders |
| depression | 311 | depressive disorder, not elsewhere classified |
| depression | 2960 | bipolar i disorder, single manic episode |
| depression | 2962 | major depressive disorder, single episode |
| depression | 2963 | major depressive disorder, recurrent episode |
| depression | 2964 | bipolar i disorder, most recent episode (or current) manic |
| depression | 2965 | bipolar i disorder, most recent episode (or current), depressed |
| depression | 2966 | bipolar i disorder, most recent episode (or current), mixed |
| depression | 2967 | bipolar i disorder, most recent episode (or current) unspecified |
| depression | 2968 | other and unspecified bipolar disorders |
| depression | 2969 | other and unspecified episodic mood disorder |
| depression | 3004 | dysthymic disorder |
| depression | 3091 | prolonged depressive reaction as adjustment reaction |
| depression | 3110 | na |
| depression | 3111 | na |
| depression | 3112 | na |
| depression | 3113 | na |
| depression | 3119 | na |
| depression | 29600 | bipolar i disorder, single manic episode, unspecified |
| depression | 29601 | bipolar i disorder, single manic episode, mild |
| depression | 29602 | bipolar i disorder, single manic episode, moderate |
| depression | 29603 | bipolar i disorder, single manic episode, severe, without mention of psychotic behavior |
| depression | 29604 | bipolar i disorder, single manic episode, severe, specified as with psychotic behavior |
| depression | 29605 | bipolar i disorder, single manic episode, in partial or unspecified remission |
| depression | 29606 | bipolar i disorder, single manic episode, in full remission |
| depression | 29620 | major depressive disorder, single episode, unspecified |
| depression | 29621 | major depressive disorder, single episode, mild |
| depression | 29622 | major depressive disorder, single episode, moderate |
| depression | 29623 | major depressive disorder, single episode, severe, without mention of psychotic behavior |
| depression | 29624 | major depressive disorder, single episode, severe, specified as with psychotic behavior |
| depression | 29625 | major depressive disorder, single episode, in partial or unspecified remission |
| depression | 29626 | major depressive disorder, single episode in full remission |
| depression | 29628 | na |
| depression | 29630 | major depressive disorder, recurrent episode, unspecified |
| depression | 29631 | major depressive disorder, recurrent episode, mild |
| depression | 29632 | major depressive disorder, recurrent episode, moderate |
| depression | 29633 | major depressive disorder, recurrent episode, severe, without mention of psychotic behavior |
| depression | 29634 | major depressive disorder, recurrent episode, severe, specified as with psychotic behavior |
| depression | 29635 | major depressive disorder, recurrent episode, in partial or unspecified remission |
| depression | 29636 | major depressive disorder, recurrent episode, in full remission |
| depression | 29637 | na |
| depression | 29640 | bipolar i disorder, most recent episode (or current) manic, unspecified |
| depression | 29641 | bipolar i disorder, most recent episode (or current) manic, mild |
| depression | 29642 | bipolar i disorder, most recent episode (or current) manic, moderate |
| depression | 29643 | bipolar i disorder, most recent episode (or current) manic, severe, without mention of psychotic behavior |
| depression | 29644 | bipolar i disorder, most recent episode (or current) manic, severe, specified as with psychotic behavior |
| depression | 29645 | bipolar i disorder, most recent episode (or current) manic, in partial or unspecified remission |
| depression | 29646 | bipolar i disorder, most recent episode (or current) manic, in full remission |
| depression | 29649 | na |
| depression | 29650 | bipolar i disorder, most recent episode (or current) depressed, unspecified |
| depression | 29651 | bipolar i disorder, most recent episode (or current) depressed, mild |
| depression | 29652 | bipolar i disorder, most recent episode (or current) depressed, moderate |
| depression | 29653 | bipolar i disorder, most recent episode (or current) depressed, severe, without mention of psychotic behavior |
| depression | 29654 | bipolar i disorder, most recent episode (or current) depressed, severe, specified as with psychotic behavior |
| depression | 29655 | bipolar i disorder, most recent episode (or current) depressed, in partial or unspecified remission |
| depression | 29656 | bipolar i disorder, most recent episode (or current) depressed, in full remission |
| depression | 29660 | bipolar i disorder, most recent episode (or current) mixed, unspecified |
| depression | 29661 | bipolar i disorder, most recent episode (or current) mixed, mild |
| depression | 29662 | bipolar i disorder, most recent episode (or current) mixed, moderate |
| depression | 29663 | bipolar i disorder, most recent episode (or current) mixed, severe, without mention of psychotic behavior |
| depression | 29664 | bipolar i disorder, most recent episode (or current) mixed, severe, specified as with psychotic behavior |
| depression | 29665 | bipolar i disorder, most recent episode (or current) mixed, in partial or unspecified remission |
| depression | 29666 | bipolar i disorder, most recent episode (or current) mixed, in full remission |
| depression | 29667 | na |
| depression | 29670 | na |
| depression | 29674 | na |
| depression | 29675 | na |
| depression | 29680 | bipolar disorder, unspecified |
| depression | 29682 | atypical depressive disorder |
| depression | 29689 | other and unspecified bipolar disorders |
| depression | 29690 | unspecified episodic mood disorder |
| depression | 29692 | na |
| depression | 29696 | na |
| depression | 29699 | other specified episodic mood disorder |
| depression | 30040 | na |
| depression | 30041 | na |
| depression | 30042 | na |
| depression | 30043 | na |
| depression | 30044 | na |
| depression | 30112 | chronic depressive personality disorder |
| depression | 30113 | cyclothymic disorder |
| depression | 30910 | na |
| depression | 30918 | na |
| depression | 31100 | na |
| depression | 31101 | na |
| depression | 31127 | na |
| depression | 31129 | na |
| depression | 31130 | na |
| depression | 31137 | na |
| depression | 31172 | na |
| depression | 31190 | na |
| depression | 311388 | na |
| depression | 296D9 | na |
| depression | 3091D | na |
| depression | 311\ | na |
| depression | 311D | na |
| depression | E95 | late effects of self-inflicted injury |
| depression | E950 | suicide and self-inflicted poisn solid/liquid sbstnc |
| depression | E9500 | suicide and self-inflicted poisoning by analgesics, antipyretics, and antirheumatics |
| depression | E9501 | suicide and self-inflicted poisoning barbiturates |
| depression | E9502 | suicide and self-inflicted poisn oth sedat and hypnot |
| depression | E9503 | suicide and self-inflicted poisoning by tranquilizers and other psychotropic agents |
| depression | E9504 | suicide and self-inflicted poisoning by other specified drugs and medicinal substances |
| depression | E9505 | suicide and self-inflicted poisoning by unspecified drug or medicinal substance |
| depression | E9506 | suicide and self-inflicted poisoning by agricultural and horticultural chemical and pharmaceutical preparations other than plant foods and fertilizers |
| depression | E9507 | suicide and self-inflicted poisoning by corrosive and caustic substances |
| depression | E9508 | suicide and self-inflicted poisn arsenic and its compnd |
| depression | E9509 | suicide and self-inflict poisn uns solid and lqd sbstnc |
| depression | E951 | suicide and self-inflicted poisoning by gases in domestic use |
| depression | E9510 | suicide and self-inflicted poisoning by gas distributed by pipeline |
| depression | E9511 | suicide and self-inflicted poisoning by liquefied petroleum gas distributed in mobile containers |
| depression | E9518 | suicide and self-inflicted poisoning by other utility gas |
| depression | E952 | suicide and self-inflicted poisn oth gases and vapors |
| depression | E9520 | suicide and self-inflict poisn motor veh exhaust gas |
| depression | E9521 | suicide and self-inflicted poisn oth carb monoxide |
| depression | E9528 | suicide and self-inflicted poisoning by other specified gases and vapors |
| depression | E9529 | suicide and self-inflicted poisoning by unspecified gases and vapors |
| depression | E953 | suicide and self-inflicted injury by hanging, strangulation, and suffocation |
| depression | E9530 | suicide and self-inflicted injury by hanging |
| depression | E9531 | suicide and self-inflicted injury by suffocation by plastic bag |
| depression | E9538 | suicide and self-inflicted injury other spec means |
| depression | E9539 | suicide and self-inflicted injury unspecified means |
| depression | E954 | suicide and self-inflicted injury by submersion (drowning) |
| depression | E955 | suicide and self-inflicted injury by firearms, air guns and explosives |
| depression | E9550 | suicide and self-inflicted injury by handgun |
| depression | E9551 | suicide and self-inflicted injury by shotgun |
| depression | E9552 | suicide and self-inflicted injury by hunting rifle |
| depression | E9553 | suicide and self-inflicted injury by military firearms |
| depression | E9554 | suicide and self-inflicted injury by other and unspecified firearm |
| depression | E9555 | suicide and self-inflicted injury by explosives |
| depression | E9556 | suicide and self-inflicted injury by air gun |
| depression | E9557 | suicide and self-inflicted injury by paintball gun |
| depression | E9559 | suicide and self-inflicted injury by firearms and explosives, unspecified |
| depression | E956 | suicide and self-inflict injury cut and piercing instrum |
| depression | E957 | suicide and self-inflicted injuries by jumping from high place |
| depression | E9570 | suicide and self-inflicted injuries by jumping from residential premises |
| depression | E9571 | suicide and slf-inflict injr jump oth man-made strct |
| depression | E9572 | suicide and self-inflicted injuries by jumping from natural sites |
| depression | E9579 | suicide and self-inflicted injuries by jumping from unspecified site |
| depression | E958 | suicide and self-inflicted injury other and unspec means |
| depression | E9580 | suicide and self-inflicted injury by jumping or lying before moving object |
| depression | E9581 | suicide and self-inflicted injury by burns, fire |
| depression | E9582 | suicide and self-inflicted injury by scald |
| depression | E9583 | suicide and self-inflicted injury by extremes of cold |
| depression | E9584 | suicide and self-inflicted injury electrocution |
| depression | E9585 | suicide and self-inflicted injury crashing motor veh |
| depression | E9586 | suicide and self-inflicted injury by crashing of aircraft |
| depression | E9587 | suicide and slf-inflict injr caustic sbstnc no poisn |
| depression | E9588 | suicide and self-inflicted injury other spec means |
| depression | E9589 | suicide and self-inflicted injury unspecified means |
| depression | E959 | late effects of self-inflicted injury |
| depression | F309 | manic episode, unspecified |
| depression | F310 | bipolar disorder, current episode hypomanic |
| depression | F3110 | bipolar disorder, current episode manic without psychotic features, unspecified |
| depression | F3111 | bipolar disorder, current episode manic without psychotic features, mild |
| depression | F3112 | bipolar disorder, current episode manic without psychotic features, moderate |
| depression | F3113 | bipolar disorder, current episode manic without psychotic features, severe |
| depression | F312 | bipolar disorder, current episode manic severe with psychotic features |
| depression | F3130 | bipolar disorder, current episode depressed, mild or moderate severity, unspecified |
| depression | F3131 | bipolar disorder, current episode depressed, mild |
| depression | F3132 | bipolar disorder, current episode depressed, moderate |
| depression | F314 | bipolar disorder, current episode depressed, severe, without psychotic features |
| depression | F315 | bipolar disorder, current episode depressed, severe, with psychotic features |
| depression | F3160 | bipolar disorder, current episode mixed, unspecified |
| depression | F3161 | bipolar disorder, current episode mixed, mild |
| depression | F3162 | bipolar disorder, current episode mixed, moderate |
| depression | F3163 | bipolar disorder, current episode mixed, severe, without psychotic features |
| depression | F3164 | bipolar disorder, current episode mixed, severe, with psychotic features |
| depression | F3170 | bipolar disorder, currently in remission, most recent episode unspecified |
| depression | F3171 | bipolar disorder, in partial remission, most recent episode hypomanic |
| depression | F3172 | bipolar disorder, in full remission, most recent episode hypomanic |
| depression | F3173 | bipolar disorder, in partial remission, most recent episode manic |
| depression | F3174 | bipolar disorder, in full remission, most recent episode manic |
| depression | F3175 | bipolar disorder, in partial remission, most recent episode depressed |
| depression | F3176 | bipolar disorder, in full remission, most recent episode depressed |
| depression | F3177 | bipolar disorder, in partial remission, most recent episode mixed |
| depression | F3178 | bipolar disorder, in full remission, most recent episode mixed |
| depression | F3181 | bipolar ii disorder |
| depression | F3189 | other bipolar disorder |
| depression | F319 | bipolar disorder, unspecified |
| depression | F320 | major depressive disorder, single episode, mild |
| depression | F321 | major depressive disorder, single episode, moderate |
| depression | F322 | major depressive disorder, single episode, severe without psychotic features |
| depression | F323 | major depressive disorder, single episode, severe with psychotic features |
| depression | F324 | major depressive disorder, single episode, in partial remission |
| depression | F325 | major depressive disorder, single episode, in full remission |
| depression | F328 | other depressive episodes |
| depression | F3289 | other specified depressive episodes |
| depression | F329 | major depressive disorder, single episode, unspecified |
| depression | F330 | major depressive disorder, recurrent, mild |
| depression | F331 | major depressive disorder, recurrent, moderate |
| depression | F332 | major depressive disorder, recurrent severe without psychotic features |
| depression | F333 | major depressive disorder, recurrent, severe with psychotic symptoms |
| depression | F3340 | major depressive disorder, recurrent, in remission, unspecified |
| depression | F3341 | major depressive disorder, recurrent, in partial remission |
| depression | F3342 | major depressive disorder, recurrent, in full remission |
| depression | F338 | other recurrent depressive disorders |
| depression | F339 | major depressive disorder, recurrent, unspecified |
| depression | F340 | cyclothymic disorder |
| depression | F341 | dysthymic disorder |
| depression | F348 | other persistent mood [affective] disorders |
| depression | F349 | persistent mood [affective] disorder, unspecified |
| depression | F39 | unspecified mood [affective] disorder |
| depression | V111 | personal history of affective disorder |
| depression | X710XXA | intentional self-harm by drowning and submersion while in bathtub, initial encounter |
| depression | X710XXD | intentional self-harm by drowning and submersion while in bathtub, subsequent encounter |
| depression | X710XXS | intentional self-harm by drowning and submersion while in bathtub, sequela |
| depression | X711XXA | intentional self-harm by drowning and submersion while in swimming pool, initial encounter |
| depression | X711XXD | intentional self-harm by drowning and submersion while in swimming pool, subsequent encounter |
| depression | X711XXS | intentional self-harm by drowning and submersion while in swimming pool, sequela |
| depression | X712XXA | intentional self-harm by drowning and submersion after jump into swimming pool, initial encounter |
| depression | X712XXD | intentional self-harm by drowning and submersion after jump into swimming pool, subsequent encounter |
| depression | X712XXS | intentional self-harm by drowning and submersion after jump into swimming pool, sequela |
| depression | X713XXA | intentional self-harm by drowning and submersion in natural water, initial encounter |
| depression | X713XXD | intentional self-harm by drowning and submersion in natural water, subsequent encounter |
| depression | X713XXS | intentional self-harm by drowning and submersion in natural water, sequela |
| depression | X718XXA | other intentional self-harm by drowning and submersion, initial encounter |
| depression | X718XXD | other intentional self-harm by drowning and submersion, subsequent encounter |
| depression | X718XXS | other intentional self-harm by drowning and submersion, sequela |
| depression | X719XXA | intentional self-harm by drowning and submersion, unspecified, initial encounter |
| depression | X719XXD | intentional self-harm by drowning and submersion, unspecified, subsequent encounter |
| depression | X719XXS | intentional self-harm by drowning and submersion, unspecified, sequela |
| depression | X72XXXA | intentional self-harm by handgun discharge, initial encounter |
| depression | X72XXXD | intentional self-harm by handgun discharge, subsequent encounter |
| depression | X72XXXS | intentional self-harm by handgun discharge, sequela |
| depression | X730XXA | intentional self-harm by shotgun discharge, initial encounter |
| depression | X730XXD | intentional self-harm by shotgun discharge, subsequent encounter |
| depression | X730XXS | intentional self-harm by shotgun discharge, sequela |
| depression | X731XXA | intentional self-harm by hunting rifle discharge, initial encounter |
| depression | X731XXD | intentional self-harm by hunting rifle discharge, subsequent encounter |
| depression | X731XXS | intentional self-harm by hunting rifle discharge, sequela |
| depression | X732XXA | intentional self-harm by machine gun discharge, initial encounter |
| depression | X732XXD | intentional self-harm by machine gun discharge, subsequent encounter |
| depression | X732XXS | intentional self-harm by machine gun discharge, sequela |
| depression | X738XXA | intentional self-harm by other larger firearm discharge, initial encounter |
| depression | X738XXD | intentional self-harm by other larger firearm discharge, subsequent encounter |
| depression | X738XXS | intentional self-harm by other larger firearm discharge, sequela |
| depression | X739XXA | intentional self-harm by unspecified larger firearm discharge, initial encounter |
| depression | X739XXD | intentional self-harm by unspecified larger firearm discharge, subsequent encounter |
| depression | X739XXS | intentional self-harm by unspecified larger firearm discharge, sequela |
| depression | X7401XA | intentional self-harm by airgun, initial encounter |
| depression | X7401XD | intentional self-harm by airgun, subsequent encounter |
| depression | X7401XS | intentional self-harm by airgun, sequela |
| depression | X7402XA | intentional self-harm by paintball gun, initial encounter |
| depression | X7402XD | intentional self-harm by paintball gun, subsequent encounter |
| depression | X7402XS | intentional self-harm by paintball gun, sequela |
| depression | X7409XA | intentional self-harm by other gas, air or spring-operated gun, initial encounter |
| depression | X7409XD | intentional self-harm by other gas, air or spring-operated gun, subsequent encounter |
| depression | X7409XS | intentional self-harm by other gas, air or spring-operated gun, sequela |
| depression | X748XXA | intentional self-harm by other firearm discharge, initial encounter |
| depression | X748XXD | intentional self-harm by other firearm discharge, subsequent encounter |
| depression | X748XXS | intentional self-harm by other firearm discharge, sequela |
| depression | X749XXA | intentional self-harm by unspecified firearm discharge, initial encounter |
| depression | X749XXD | intentional self-harm by unspecified firearm discharge, subsequent encounter |
| depression | X749XXS | intentional self-harm by unspecified firearm discharge, sequela |
| depression | X75XXXA | intentional self-harm by explosive material, initial encounter |
| depression | X75XXXD | intentional self-harm by explosive material, subsequent encounter |
| depression | X75XXXS | intentional self-harm by explosive material, sequela |
| depression | X76XXXA | intentional self-harm by smoke, fire and flames, initial encounter |
| depression | X76XXXD | intentional self-harm by smoke, fire and flames, subsequent encounter |
| depression | X76XXXS | intentional self-harm by smoke, fire and flames, sequela |
| depression | X770XXA | intentional self-harm by steam or hot vapors, initial encounter |
| depression | X770XXD | intentional self-harm by steam or hot vapors, subsequent encounter |
| depression | X770XXS | intentional self-harm by steam or hot vapors, sequela |
| depression | X771XXA | intentional self-harm by hot tap water, initial encounter |
| depression | X771XXD | intentional self-harm by hot tap water, subsequent encounter |
| depression | X771XXS | intentional self-harm by hot tap water, sequela |
| depression | X772XXA | intentional self-harm by other hot fluids, initial encounter |
| depression | X772XXD | intentional self-harm by other hot fluids, subsequent encounter |
| depression | X772XXS | intentional self-harm by other hot fluids, sequela |
| depression | X773XXA | intentional self-harm by hot household appliances, initial encounter |
| depression | X773XXD | intentional self-harm by hot household appliances, subsequent encounter |
| depression | X773XXS | intentional self-harm by hot household appliances, sequela |
| depression | X778XXA | intentional self-harm by other hot objects, initial encounter |
| depression | X778XXD | intentional self-harm by other hot objects, subsequent encounter |
| depression | X778XXS | intentional self-harm by other hot objects, sequela |
| depression | X779XXA | intentional self-harm by unspecified hot objects, initial encounter |
| depression | X779XXD | intentional self-harm by unspecified hot objects, subsequent encounter |
| depression | X779XXS | intentional self-harm by unspecified hot objects, sequela |
| depression | X780XXA | intentional self-harm by sharp glass, initial encounter |
| depression | X780XXD | intentional self-harm by sharp glass, subsequent encounter |
| depression | X780XXS | intentional self-harm by sharp glass, sequela |
| depression | X781XXA | intentional self-harm by knife, initial encounter |
| depression | X781XXD | intentional self-harm by knife, subsequent encounter |
| depression | X781XXS | intentional self-harm by knife, sequela |
| depression | X782XXA | intentional self-harm by sword or dagger, initial encounter |
| depression | X782XXD | intentional self-harm by sword or dagger, subsequent encounter |
| depression | X782XXS | intentional self-harm by sword or dagger, sequela |
| depression | X788XXA | intentional self-harm by other sharp object, initial encounter |
| depression | X788XXD | intentional self-harm by other sharp object, subsequent encounter |
| depression | X788XXS | intentional self-harm by other sharp object, sequela |
| depression | X789XXA | intentional self-harm by unspecified sharp object, initial encounter |
| depression | X789XXD | intentional self-harm by unspecified sharp object, subsequent encounter |
| depression | X789XXS | intentional self-harm by unspecified sharp object, sequela |
| depression | X79XXXA | intentional self-harm by blunt object, initial encounter |
| depression | X79XXXD | intentional self-harm by blunt object, subsequent encounter |
| depression | X79XXXS | intentional self-harm by blunt object, sequela |
| depression | X80XXXA | intentional self-harm by jumping from a high place, initial encounter |
| depression | X80XXXD | intentional self-harm by jumping from a high place, subsequent encounter |
| depression | X80XXXS | intentional self-harm by jumping from a high place, sequela |
| depression | X810XXA | intentional self-harm by jumping or lying in front of motor vehicle, initial encounter |
| depression | X810XXD | intentional self-harm by jumping or lying in front of motor vehicle, subsequent encounter |
| depression | X810XXS | intentional self-harm by jumping or lying in front of motor vehicle, sequela |
| depression | X811XXA | intentional self-harm by jumping or lying in front of (subway) train, initial encounter |
| depression | X811XXD | intentional self-harm by jumping or lying in front of (subway) train, subsequent encounter |
| depression | X811XXS | intentional self-harm by jumping or lying in front of (subway) train, sequela |
| depression | X818XXA | intentional self-harm by jumping or lying in front of other moving object, initial encounter |
| depression | X818XXD | intentional self-harm by jumping or lying in front of other moving object, subsequent encounter |
| depression | X818XXS | intentional self-harm by jumping or lying in front of other moving object, sequela |
| depression | X820XXA | intentional collision of motor vehicle with other motor vehicle, initial encounter |
| depression | X820XXD | intentional collision of motor vehicle with other motor vehicle, subsequent encounter |
| depression | X820XXS | intentional collision of motor vehicle with other motor vehicle, sequela |
| depression | X821XXA | intentional collision of motor vehicle with train, initial encounter |
| depression | X821XXD | intentional collision of motor vehicle with train, subsequent encounter |
| depression | X821XXS | intentional collision of motor vehicle with train, sequela |
| depression | X822XXA | intentional collision of motor vehicle with tree, initial encounter |
| depression | X822XXD | intentional collision of motor vehicle with tree, subsequent encounter |
| depression | X822XXS | intentional collision of motor vehicle with tree, sequela |
| depression | X828XXA | other intentional self-harm by crashing of motor vehicle, initial encounter |
| depression | X828XXD | other intentional self-harm by crashing of motor vehicle, subsequent encounter |
| depression | X828XXS | other intentional self-harm by crashing of motor vehicle, sequela |
| depression | X830XXA | intentional self-harm by crashing of aircraft, initial encounter |
| depression | X830XXD | intentional self-harm by crashing of aircraft, subsequent encounter |
| depression | X830XXS | intentional self-harm by crashing of aircraft, sequela |
| depression | X831XXA | intentional self-harm by electrocution, initial encounter |
| depression | X831XXD | intentional self-harm by electrocution, subsequent encounter |
| depression | X831XXS | intentional self-harm by electrocution, sequela |
| depression | X832XXA | intentional self-harm by exposure to extremes of cold, initial encounter |
| depression | X832XXD | intentional self-harm by exposure to extremes of cold, subsequent encounter |
| depression | X832XXS | intentional self-harm by exposure to extremes of cold, sequela |
| depression | X838XXA | intentional self-harm by other specified means, initial encounter |
| depression | X838XXD | intentional self-harm by other specified means, subsequent encounter |
| depression | X838XXS | intentional self-harm by other specified means, sequela |
| diabetes | 25 | na |
| diabetes | 249 | secondary diabetes mellitus |
| diabetes | 250 | diabetes mellitus |
| diabetes | 2490 | secondary diabetes mellitus without mention of complication |
| diabetes | 2491 | secondary diabetes mellitus with ketoacidosis |
| diabetes | 2492 | secondary diabetes mellitus with hyperosmolarity |
| diabetes | 2493 | secondary diabetes mellitus with other coma |
| diabetes | 2494 | secondary diabetes mellitus with renal manifestations |
| diabetes | 2495 | secondary diabetes mellitus with ophthalmic manifestations |
| diabetes | 2496 | secondary diabetes mellitus with neurological manifestations |
| diabetes | 2497 | secondary diabetes mellitus with peripheral circulatory disorders |
| diabetes | 2498 | secondary diabetes mellitus with other specified manifestations |
| diabetes | 2499 | secondary diabetes mellitus with unspecified complication |
| diabetes | 2500 | diabetes mellitus without mention of complication |
| diabetes | 2501 | diabetes with ketoacidosis |
| diabetes | 2502 | diabetes with hyperosmolarity |
| diabetes | 2503 | diabetes with other coma |
| diabetes | 2504 | diabetes with renal manifestations |
| diabetes | 2505 | diabetes with ophthalmic manifestations |
| diabetes | 2506 | diabetes with neurological manifestations |
| diabetes | 2507 | diabetes with peripheral circulatory disorders |
| diabetes | 2508 | diabetes with other specified manifestations |
| diabetes | 2509 | diabetes with unspecified complication |
| diabetes | 3572 | polyneuropathy in diabetes |
| diabetes | 3620 | diabetic retinopathy |
| diabetes | 6480 | maternal diabetes mellitus complicating pregnancy, childbirth, or the puerperium |
| diabetes | 24690 | na |
| diabetes | 24900 | secondary diabetes mellitus without mention of complication, not stated as uncontrolled, or unspecified |
| diabetes | 24901 | secondary diabetes mellitus without mention of complication, uncontrolled |
| diabetes | 24910 | secondary diabetes mellitus with ketoacidosis, not stated as uncontrolled, or unspecified |
| diabetes | 24911 | secondary diabetes mellitus with ketoacidosis, uncontrolled |
| diabetes | 24920 | secondary diabetes mellitus with hyperosmolarity, not stated as uncontrolled, or unspecified |
| diabetes | 24921 | secondary diabetes mellitus with hyperosmolarity, uncontrolled |
| diabetes | 24930 | secondary diabetes mellitus with other coma, not stated as uncontrolled, or unspecified |
| diabetes | 24931 | secondary diabetes mellitus with other coma, uncontrolled |
| diabetes | 24940 | secondary diabetes mellitus with renal manifestations, not stated as uncontrolled, or unspecified |
| diabetes | 24941 | secondary diabetes mellitus with renal manifestations, uncontrolled |
| diabetes | 24950 | secondary diabetes mellitus with ophthalmic manifestations, not stated as uncontrolled, or unspecified |
| diabetes | 24951 | secondary diabetes mellitus with ophthalmic manifestations, uncontrolled |
| diabetes | 24960 | secondary diabetes mellitus with neurological manifestations, not stated as uncontrolled, or unspecified |
| diabetes | 24961 | secondary diabetes mellitus with neurological manifestations, uncontrolled |
| diabetes | 24970 | secondary diabetes mellitus with peripheral circulatory disorders, not stated as uncontrolled, or unspecified |
| diabetes | 24971 | secondary diabetes mellitus with peripheral circulatory disorders, uncontrolled |
| diabetes | 24980 | secondary diabetes mellitus with other specified manifestations, not stated as uncontrolled, or unspecified |
| diabetes | 24981 | secondary diabetes mellitus with other specified manifestations, uncontrolled |
| diabetes | 24990 | secondary diabetes mellitus with unspecified complication, not stated as uncontrolled, or unspecified |
| diabetes | 24991 | secondary diabetes mellitus with unspecified complication, uncontrolled |
| diabetes | 25000 | diabetes mellitus without mention of complication, type ii or unspecified type, not stated as uncontrolled |
| diabetes | 25001 | diabetes mellitus without mention of complication, type i [juvenile type], not stated as uncontrolled |
| diabetes | 25002 | diabetes mellitus without mention of complication, type ii or unspecified type, uncontrolled |
| diabetes | 25003 | diabetes mellitus without mention of complication, type i [juvenile type], uncontrolled |
| diabetes | 25005 | na |
| diabetes | 25006 | na |
| diabetes | 25007 | na |
| diabetes | 25008 | na |
| diabetes | 25009 | na |
| diabetes | 25010 | diabetes with ketoacidosis, type ii or unspecified type, not stated as uncontrolled |
| diabetes | 25011 | diabetes with ketoacidosis, type i [juvenile type], not stated as uncontrolled |
| diabetes | 25012 | diabetes with ketoacidosis, type ii or unspecified type, uncontrolled |
| diabetes | 25013 | diabetes with ketoacidosis, type i [juvenile type], uncontrolled |
| diabetes | 25020 | diabetes with hyperosmolarity, type ii or unspecified type, not stated as uncontrolled |
| diabetes | 25021 | diabetes with hyperosmolarity, type i [juvenile type], not stated as uncontrolled |
| diabetes | 25022 | diabetes with hyperosmolarity, type ii or unspecified type, uncontrolled |
| diabetes | 25023 | diabetes with hyperosmolarity, type i [juvenile type], uncontrolled |
| diabetes | 25025 | na |
| diabetes | 25028 | na |
| diabetes | 25030 | diabetes with other coma, type ii or unspecified type, not stated as uncontrolled |
| diabetes | 25031 | diabetes with other coma, type i [juvenile type], not stated as uncontrolled |
| diabetes | 25032 | diabetes with other coma, type ii or unspecified type, uncontrolled |
| diabetes | 25033 | diabetes with other coma, type i [juvenile type], uncontrolled |
| diabetes | 25040 | diabetes with renal manifestations, type ii or unspecified type, not stated as uncontrolled |
| diabetes | 25041 | diabetes with renal manifestations, type i [juvenile type], not stated as uncontrolled |
| diabetes | 25042 | diabetes with renal manifestations, type ii or unspecified type, uncontrolled |
| diabetes | 25043 | diabetes with renal manifestations, type i [juvenile type], uncontrolled |
| diabetes | 25048 | na |
| diabetes | 25050 | diabetes with ophthalmic manifestations, type ii or unspecified type, not stated as uncontrolled |
| diabetes | 25051 | diabetes with ophthalmic manifestations, type i [juvenile type], not stated as uncontrolled |
| diabetes | 25052 | diabetes with ophthalmic manifestations, type ii or unspecified type, uncontrolled |
| diabetes | 25053 | diabetes with ophthalmic manifestations, type i [juvenile type], uncontrolled |
| diabetes | 25058 | na |
| diabetes | 25060 | diabetes with neurological manifestations, type ii or unspecified type, not stated as uncontrolled |
| diabetes | 25061 | diabetes with neurological manifestations, type i [juvenile type], not stated as uncontrolled |
| diabetes | 25062 | diabetes with neurological manifestations, type ii or unspecified type, uncontrolled |
| diabetes | 25063 | diabetes with neurological manifestations, type i [juvenile type], uncontrolled |
| diabetes | 25067 | na |
| diabetes | 25068 | na |
| diabetes | 25070 | diabetes with peripheral circulatory disorders, type ii or unspecified type, not stated as uncontrolled |
| diabetes | 25071 | diabetes with peripheral circulatory disorders, type i [juvenile type], not stated as uncontrolled |
| diabetes | 25072 | diabetes with peripheral circulatory disorders, type ii or unspecified type, uncontrolled |
| diabetes | 25073 | diabetes with peripheral circulatory disorders, type i [juvenile type], uncontrolled |
| diabetes | 25080 | diabetes with other specified manifestations, type ii or unspecified type, not stated as uncontrolled |
| diabetes | 25081 | diabetes with other specified manifestations, type i [juvenile type], not stated as uncontrolled |
| diabetes | 25082 | diabetes with other specified manifestations, type ii or unspecified type, uncontrolled |
| diabetes | 25083 | diabetes with other specified manifestations, type i [juvenile type], uncontrolled |
| diabetes | 25090 | diabetes with unspecified complication, type ii or unspecified type, not stated as uncontrolled |
| diabetes | 25091 | diabetes with unspecified complication, type i [juvenile type], not stated as uncontrolled |
| diabetes | 25092 | diabetes with unspecified complication, type ii or unspecified type, uncontrolled |
| diabetes | 25093 | diabetes with unspecified complication, type i [juvenile type], uncontrolled |
| diabetes | 25095 | na |
| diabetes | 25097 | na |
| diabetes | 35720 | na |
| diabetes | 35721 | na |
| diabetes | 35723 | na |
| diabetes | 36200 | na |
| diabetes | 36201 | background diabetic retinopathy |
| diabetes | 36202 | proliferative diabetic retinopathy |
| diabetes | 36203 | nonproliferative diabetic retinopathy nos |
| diabetes | 36204 | mild nonproliferative diabetic retinopathy |
| diabetes | 36205 | moderate nonproliferative diabetic retinopathy |
| diabetes | 36206 | severe nonproliferative diabetic retinopathy |
| diabetes | 36207 | diabetic macular edema |
| diabetes | 36641 | diabetic cataract |
| diabetes | 64800 | maternal diabetes mellitus, complicating pregnancy, childbirth, or the puerperium, unspecified as to episode of car |
| diabetes | 64801 | maternal diabetes mellitus with delivery |
| diabetes | 64802 | maternal diabetes mellitus with delivery, with current postpartum complication |
| diabetes | 64803 | maternal diabetes mellitus, antepartum |
| diabetes | 64804 | maternal diabetes mellitus, previous postpartum condition |
| diabetes | 250000 | na |
| diabetes | 250002 | na |
| diabetes | 250003 | na |
| diabetes | 250007 | na |
| diabetes | 250024 | na |
| diabetes | 250631 | na |
| diabetes | 250 2 | diabetes with hyperosmolarity |
| diabetes | E0800 | diabetes mellitus due to underlying condition with hyperosmolarity without nonketotic hyperglycemic-hyperosmolar coma (nkhhc) |
| diabetes | E0801 | diabetes mellitus due to underlying condition with hyperosmolarity with coma |
| diabetes | E0810 | diabetes mellitus due to underlying condition with ketoacidosis without coma |
| diabetes | E0811 | diabetes mellitus due to underlying condition with ketoacidosis with coma |
| diabetes | E0821 | diabetes mellitus due to underlying condition with diabetic nephropathy |
| diabetes | E0822 | diabetes mellitus due to underlying condition with diabetic chronic kidney disease |
| diabetes | E0829 | diabetes mellitus due to underlying condition with other diabetic kidney complication |
| diabetes | E08311 | diabetes mellitus due to underlying condition with unspecified diabetic retinopathy with macular edema |
| diabetes | E08319 | diabetes mellitus due to underlying condition with unspecified diabetic retinopathy without macular edema |
| diabetes | E08321 | diabetes mellitus due to underlying condition with mild nonproliferative diabetic retinopathy with macular edema |
| diabetes | E083211 | diabetes mellitus due to underlying condition with mild nonproliferative diabetic retinopathy with macular edema, right eye |
| diabetes | E083212 | diabetes mellitus due to underlying condition with mild nonproliferative diabetic retinopathy with macular edema, left eye |
| diabetes | E083213 | diabetes mellitus due to underlying condition with mild nonproliferative diabetic retinopathy with macular edema, bilateral |
| diabetes | E083219 | diabetes mellitus due to underlying condition with mild nonproliferative diabetic retinopathy with macular edema, unspecified eye |
| diabetes | E08329 | diabetes mellitus due to underlying condition with mild nonproliferative diabetic retinopathy without macular edema |
| diabetes | E083291 | diabetes mellitus due to underlying condition with mild nonproliferative diabetic retinopathy without macular edema, right eye |
| diabetes | E083292 | diabetes mellitus due to underlying condition with mild nonproliferative diabetic retinopathy without macular edema, left eye |
| diabetes | E083293 | diabetes mellitus due to underlying condition with mild nonproliferative diabetic retinopathy without macular edema, bilateral |
| diabetes | E083299 | diabetes mellitus due to underlying condition with mild nonproliferative diabetic retinopathy without macular edema, unspecified eye |
| diabetes | E08331 | diabetes mellitus due to underlying condition with moderate nonproliferative diabetic retinopathy with macular edema |
| diabetes | E083311 | diabetes mellitus due to underlying condition with moderate nonproliferative diabetic retinopathy with macular edema, right eye |
| diabetes | E083312 | diabetes mellitus due to underlying condition with moderate nonproliferative diabetic retinopathy with macular edema, left eye |
| diabetes | E083313 | diabetes mellitus due to underlying condition with moderate nonproliferative diabetic retinopathy with macular edema, bilateral |
| diabetes | E083319 | diabetes mellitus due to underlying condition with moderate nonproliferative diabetic retinopathy with macular edema, unspecified eye |
| diabetes | E08339 | diabetes mellitus due to underlying condition with moderate nonproliferative diabetic retinopathy without macular edema |
| diabetes | E083391 | diabetes mellitus due to underlying condition with moderate nonproliferative diabetic retinopathy without macular edema, right eye |
| diabetes | E083392 | diabetes mellitus due to underlying condition with moderate nonproliferative diabetic retinopathy without macular edema, left eye |
| diabetes | E083393 | diabetes mellitus due to underlying condition with moderate nonproliferative diabetic retinopathy without macular edema, bilateral |
| diabetes | E083399 | diabetes mellitus due to underlying condition with moderate nonproliferative diabetic retinopathy without macular edema, unspecified eye |
| diabetes | E08341 | diabetes mellitus due to underlying condition with severe nonproliferative diabetic retinopathy with macular edema |
| diabetes | E083411 | diabetes mellitus due to underlying condition with severe nonproliferative diabetic retinopathy with macular edema, right eye |
| diabetes | E083412 | diabetes mellitus due to underlying condition with severe nonproliferative diabetic retinopathy with macular edema, left eye |
| diabetes | E083413 | diabetes mellitus due to underlying condition with severe nonproliferative diabetic retinopathy with macular edema, bilateral |
| diabetes | E083419 | diabetes mellitus due to underlying condition with severe nonproliferative diabetic retinopathy with macular edema, unspecified eye |
| diabetes | E08349 | diabetes mellitus due to underlying condition with severe nonproliferative diabetic retinopathy without macular edema |
| diabetes | E083491 | diabetes mellitus due to underlying condition with severe nonproliferative diabetic retinopathy without macular edema, right eye |
| diabetes | E083492 | diabetes mellitus due to underlying condition with severe nonproliferative diabetic retinopathy without macular edema, left eye |
| diabetes | E083493 | diabetes mellitus due to underlying condition with severe nonproliferative diabetic retinopathy without macular edema, bilateral |
| diabetes | E083499 | diabetes mellitus due to underlying condition with severe nonproliferative diabetic retinopathy without macular edema, unspecified eye |
| diabetes | E08351 | diabetes mellitus due to underlying condition with proliferative diabetic retinopathy with macular edema |
| diabetes | E083511 | diabetes mellitus due to underlying condition with proliferative diabetic retinopathy with macular edema, right eye |
| diabetes | E083512 | diabetes mellitus due to underlying condition with proliferative diabetic retinopathy with macular edema, left eye |
| diabetes | E083513 | diabetes mellitus due to underlying condition with proliferative diabetic retinopathy with macular edema, bilateral |
| diabetes | E083519 | diabetes mellitus due to underlying condition with proliferative diabetic retinopathy with macular edema, unspecified eye |
| diabetes | E083521 | diabetes mellitus due to underlying condition with proliferative diabetic retinopathy with traction retinal detachment involving the macula, right eye |
| diabetes | E083522 | diabetes mellitus due to underlying condition with proliferative diabetic retinopathy with traction retinal detachment involving the macula, left eye |
| diabetes | E083523 | diabetes mellitus due to underlying condition with proliferative diabetic retinopathy with traction retinal detachment involving the macula, bilateral |
| diabetes | E083529 | diabetes mellitus due to underlying condition with proliferative diabetic retinopathy with traction retinal detachment involving the macula, unspecified eye |
| diabetes | E083531 | diabetes mellitus due to underlying condition with proliferative diabetic retinopathy with traction retinal detachment not involving the macula, right eye |
| diabetes | E083532 | diabetes mellitus due to underlying condition with proliferative diabetic retinopathy with traction retinal detachment not involving the macula, left eye |
| diabetes | E083533 | diabetes mellitus due to underlying condition with proliferative diabetic retinopathy with traction retinal detachment not involving the macula, bilateral |
| diabetes | E083539 | diabetes mellitus due to underlying condition with proliferative diabetic retinopathy with traction retinal detachment not involving the macula, unspecified eye |
| diabetes | E083541 | diabetes mellitus due to underlying condition with proliferative diabetic retinopathy with combined traction retinal detachment and rhegmatogenous retinal detachment, right eye |
| diabetes | E083542 | diabetes mellitus due to underlying condition with proliferative diabetic retinopathy with combined traction retinal detachment and rhegmatogenous retinal detachment, left eye |
| diabetes | E083543 | diabetes mellitus due to underlying condition with proliferative diabetic retinopathy with combined traction retinal detachment and rhegmatogenous retinal detachment, bilateral |
| diabetes | E083549 | diabetes mellitus due to underlying condition with proliferative diabetic retinopathy with combined traction retinal detachment and rhegmatogenous retinal detachment, unspecified eye |
| diabetes | E083551 | diabetes mellitus due to underlying condition with stable proliferative diabetic retinopathy, right eye |
| diabetes | E083552 | diabetes mellitus due to underlying condition with stable proliferative diabetic retinopathy, left eye |
| diabetes | E083553 | diabetes mellitus due to underlying condition with stable proliferative diabetic retinopathy, bilateral |
| diabetes | E083559 | diabetes mellitus due to underlying condition with stable proliferative diabetic retinopathy, unspecified eye |
| diabetes | E08359 | diabetes mellitus due to underlying condition with proliferative diabetic retinopathy without macular edema |
| diabetes | E083591 | diabetes mellitus due to underlying condition with proliferative diabetic retinopathy without macular edema, right eye |
| diabetes | E083592 | diabetes mellitus due to underlying condition with proliferative diabetic retinopathy without macular edema, left eye |
| diabetes | E083593 | diabetes mellitus due to underlying condition with proliferative diabetic retinopathy without macular edema, bilateral |
| diabetes | E083599 | diabetes mellitus due to underlying condition with proliferative diabetic retinopathy without macular edema, unspecified eye |
| diabetes | E0836 | diabetes mellitus due to underlying condition with diabetic cataract |
| diabetes | E0837X1 | diabetes mellitus due to underlying condition with diabetic macular edema, resolved following treatment, right eye |
| diabetes | E0837X2 | diabetes mellitus due to underlying condition with diabetic macular edema, resolved following treatment, left eye |
| diabetes | E0837X3 | diabetes mellitus due to underlying condition with diabetic macular edema, resolved following treatment, bilateral |
| diabetes | E0837X9 | diabetes mellitus due to underlying condition with diabetic macular edema, resolved following treatment, unspecified eye |
| diabetes | E0839 | diabetes mellitus due to underlying condition with other diabetic ophthalmic complication |
| diabetes | E0840 | diabetes mellitus due to underlying condition with diabetic neuropathy, unspecified |
| diabetes | E0841 | diabetes mellitus due to underlying condition with diabetic mononeuropathy |
| diabetes | E0842 | diabetes mellitus due to underlying condition with diabetic polyneuropathy |
| diabetes | E0843 | diabetes mellitus due to underlying condition with diabetic autonomic (poly)neuropathy |
| diabetes | E0844 | diabetes mellitus due to underlying condition with diabetic amyotrophy |
| diabetes | E0849 | diabetes mellitus due to underlying condition with other diabetic neurological complication |
| diabetes | E0851 | diabetes mellitus due to underlying condition with diabetic peripheral angiopathy without gangrene |
| diabetes | E0852 | diabetes mellitus due to underlying condition with diabetic peripheral angiopathy with gangrene |
| diabetes | E0859 | diabetes mellitus due to underlying condition with other circulatory complications |
| diabetes | E08610 | diabetes mellitus due to underlying condition with diabetic neuropathic arthropathy |
| diabetes | E08618 | diabetes mellitus due to underlying condition with other diabetic arthropathy |
| diabetes | E08620 | diabetes mellitus due to underlying condition with diabetic dermatitis |
| diabetes | E08621 | diabetes mellitus due to underlying condition with foot ulcer |
| diabetes | E08622 | diabetes mellitus due to underlying condition with other skin ulcer |
| diabetes | E08628 | diabetes mellitus due to underlying condition with other skin complications |
| diabetes | E08630 | diabetes mellitus due to underlying condition with periodontal disease |
| diabetes | E08638 | diabetes mellitus due to underlying condition with other oral complications |
| diabetes | E08641 | diabetes mellitus due to underlying condition with hypoglycemia with coma |
| diabetes | E08649 | diabetes mellitus due to underlying condition with hypoglycemia without coma |
| diabetes | E0865 | diabetes mellitus due to underlying condition with hyperglycemia |
| diabetes | E0869 | diabetes mellitus due to underlying condition with other specified complication |
| diabetes | E088 | diabetes mellitus due to underlying condition with unspecified complications |
| diabetes | E089 | diabetes mellitus due to underlying condition without complications |
| diabetes | E0900 | drug or chemical induced diabetes mellitus with hyperosmolarity without nonketotic hyperglycemic-hyperosmolar coma (nkhhc) |
| diabetes | E0901 | drug or chemical induced diabetes mellitus with hyperosmolarity with coma |
| diabetes | E0910 | drug or chemical induced diabetes mellitus with ketoacidosis without coma |
| diabetes | E0911 | drug or chemical induced diabetes mellitus with ketoacidosis with coma |
| diabetes | E0921 | drug or chemical induced diabetes mellitus with diabetic nephropathy |
| diabetes | E0922 | drug or chemical induced diabetes mellitus with diabetic chronic kidney disease |
| diabetes | E0929 | drug or chemical induced diabetes mellitus with other diabetic kidney complication |
| diabetes | E09311 | drug or chemical induced diabetes mellitus with unspecified diabetic retinopathy with macular edema |
| diabetes | E09319 | drug or chemical induced diabetes mellitus with unspecified diabetic retinopathy without macular edema |
| diabetes | E09321 | drug or chemical induced diabetes mellitus with mild nonproliferative diabetic retinopathy with macular edema |
| diabetes | E093211 | drug or chemical induced diabetes mellitus with mild nonproliferative diabetic retinopathy with macular edema, right eye |
| diabetes | E093212 | drug or chemical induced diabetes mellitus with mild nonproliferative diabetic retinopathy with macular edema, left eye |
| diabetes | E093213 | drug or chemical induced diabetes mellitus with mild nonproliferative diabetic retinopathy with macular edema, bilateral |
| diabetes | E093219 | drug or chemical induced diabetes mellitus with mild nonproliferative diabetic retinopathy with macular edema, unspecified eye |
| diabetes | E09329 | drug or chemical induced diabetes mellitus with mild nonproliferative diabetic retinopathy without macular edema |
| diabetes | E093291 | drug or chemical induced diabetes mellitus with mild nonproliferative diabetic retinopathy without macular edema, right eye |
| diabetes | E093292 | drug or chemical induced diabetes mellitus with mild nonproliferative diabetic retinopathy without macular edema, left eye |
| diabetes | E093293 | drug or chemical induced diabetes mellitus with mild nonproliferative diabetic retinopathy without macular edema, bilateral |
| diabetes | E093299 | drug or chemical induced diabetes mellitus with mild nonproliferative diabetic retinopathy without macular edema, unspecified eye |
| diabetes | E09331 | drug or chemical induced diabetes mellitus with moderate nonproliferative diabetic retinopathy with macular edema |
| diabetes | E093311 | drug or chemical induced diabetes mellitus with moderate nonproliferative diabetic retinopathy with macular edema, right eye |
| diabetes | E093312 | drug or chemical induced diabetes mellitus with moderate nonproliferative diabetic retinopathy with macular edema, left eye |
| diabetes | E093313 | drug or chemical induced diabetes mellitus with moderate nonproliferative diabetic retinopathy with macular edema, bilateral |
| diabetes | E093319 | drug or chemical induced diabetes mellitus with moderate nonproliferative diabetic retinopathy with macular edema, unspecified eye |
| diabetes | E09339 | drug or chemical induced diabetes mellitus with moderate nonproliferative diabetic retinopathy without macular edema |
| diabetes | E093391 | drug or chemical induced diabetes mellitus with moderate nonproliferative diabetic retinopathy without macular edema, right eye |
| diabetes | E093392 | drug or chemical induced diabetes mellitus with moderate nonproliferative diabetic retinopathy without macular edema, left eye |
| diabetes | E093393 | drug or chemical induced diabetes mellitus with moderate nonproliferative diabetic retinopathy without macular edema, bilateral |
| diabetes | E093399 | drug or chemical induced diabetes mellitus with moderate nonproliferative diabetic retinopathy without macular edema, unspecified eye |
| diabetes | E09341 | drug or chemical induced diabetes mellitus with severe nonproliferative diabetic retinopathy with macular edema |
| diabetes | E093411 | drug or chemical induced diabetes mellitus with severe nonproliferative diabetic retinopathy with macular edema, right eye |
| diabetes | E093412 | drug or chemical induced diabetes mellitus with severe nonproliferative diabetic retinopathy with macular edema, left eye |
| diabetes | E093413 | drug or chemical induced diabetes mellitus with severe nonproliferative diabetic retinopathy with macular edema, bilateral |
| diabetes | E093419 | drug or chemical induced diabetes mellitus with severe nonproliferative diabetic retinopathy with macular edema, unspecified eye |
| diabetes | E09349 | drug or chemical induced diabetes mellitus with severe nonproliferative diabetic retinopathy without macular edema |
| diabetes | E093491 | drug or chemical induced diabetes mellitus with severe nonproliferative diabetic retinopathy without macular edema, right eye |
| diabetes | E093492 | drug or chemical induced diabetes mellitus with severe nonproliferative diabetic retinopathy without macular edema, left eye |
| diabetes | E093493 | drug or chemical induced diabetes mellitus with severe nonproliferative diabetic retinopathy without macular edema, bilateral |
| diabetes | E093499 | drug or chemical induced diabetes mellitus with severe nonproliferative diabetic retinopathy without macular edema, unspecified eye |
| diabetes | E09351 | drug or chemical induced diabetes mellitus with proliferative diabetic retinopathy with macular edema |
| diabetes | E093511 | drug or chemical induced diabetes mellitus with proliferative diabetic retinopathy with macular edema, right eye |
| diabetes | E093512 | drug or chemical induced diabetes mellitus with proliferative diabetic retinopathy with macular edema, left eye |
| diabetes | E093513 | drug or chemical induced diabetes mellitus with proliferative diabetic retinopathy with macular edema, bilateral |
| diabetes | E093519 | drug or chemical induced diabetes mellitus with proliferative diabetic retinopathy with macular edema, unspecified eye |
| diabetes | E093521 | drug or chemical induced diabetes mellitus with proliferative diabetic retinopathy with traction retinal detachment involving the macula, right eye |
| diabetes | E093522 | drug or chemical induced diabetes mellitus with proliferative diabetic retinopathy with traction retinal detachment involving the macula, left eye |
| diabetes | E093523 | drug or chemical induced diabetes mellitus with proliferative diabetic retinopathy with traction retinal detachment involving the macula, bilateral |
| diabetes | E093529 | drug or chemical induced diabetes mellitus with proliferative diabetic retinopathy with traction retinal detachment involving the macula, unspecified eye |
| diabetes | E093531 | drug or chemical induced diabetes mellitus with proliferative diabetic retinopathy with traction retinal detachment not involving the macula, right eye |
| diabetes | E093532 | drug or chemical induced diabetes mellitus with proliferative diabetic retinopathy with traction retinal detachment not involving the macula, left eye |
| diabetes | E093533 | drug or chemical induced diabetes mellitus with proliferative diabetic retinopathy with traction retinal detachment not involving the macula, bilateral |
| diabetes | E093539 | drug or chemical induced diabetes mellitus with proliferative diabetic retinopathy with traction retinal detachment not involving the macula, unspecified eye |
| diabetes | E093541 | drug or chemical induced diabetes mellitus with proliferative diabetic retinopathy with combined traction retinal detachment and rhegmatogenous retinal detachment, right eye |
| diabetes | E093542 | drug or chemical induced diabetes mellitus with proliferative diabetic retinopathy with combined traction retinal detachment and rhegmatogenous retinal detachment, left eye |
| diabetes | E093543 | drug or chemical induced diabetes mellitus with proliferative diabetic retinopathy with combined traction retinal detachment and rhegmatogenous retinal detachment, bilateral |
| diabetes | E093549 | drug or chemical induced diabetes mellitus with proliferative diabetic retinopathy with combined traction retinal detachment and rhegmatogenous retinal detachment, unspecified eye |
| diabetes | E093551 | drug or chemical induced diabetes mellitus with stable proliferative diabetic retinopathy, right eye |
| diabetes | E093552 | drug or chemical induced diabetes mellitus with stable proliferative diabetic retinopathy, left eye |
| diabetes | E093553 | drug or chemical induced diabetes mellitus with stable proliferative diabetic retinopathy, bilateral |
| diabetes | E093559 | drug or chemical induced diabetes mellitus with stable proliferative diabetic retinopathy, unspecified eye |
| diabetes | E09359 | drug or chemical induced diabetes mellitus with proliferative diabetic retinopathy without macular edema |
| diabetes | E093591 | drug or chemical induced diabetes mellitus with proliferative diabetic retinopathy without macular edema, right eye |
| diabetes | E093592 | drug or chemical induced diabetes mellitus with proliferative diabetic retinopathy without macular edema, left eye |
| diabetes | E093593 | drug or chemical induced diabetes mellitus with proliferative diabetic retinopathy without macular edema, bilateral |
| diabetes | E093599 | drug or chemical induced diabetes mellitus with proliferative diabetic retinopathy without macular edema, unspecified eye |
| diabetes | E0936 | drug or chemical induced diabetes mellitus with diabetic cataract |
| diabetes | E0937X1 | drug or chemical induced diabetes mellitus with diabetic macular edema, resolved following treatment, right eye |
| diabetes | E0937X2 | drug or chemical induced diabetes mellitus with diabetic macular edema, resolved following treatment, left eye |
| diabetes | E0937X3 | drug or chemical induced diabetes mellitus with diabetic macular edema, resolved following treatment, bilateral |
| diabetes | E0937X9 | drug or chemical induced diabetes mellitus with diabetic macular edema, resolved following treatment, unspecified eye |
| diabetes | E0939 | drug or chemical induced diabetes mellitus with other diabetic ophthalmic complication |
| diabetes | E0940 | drug or chemical induced diabetes mellitus with neurological complications with diabetic neuropathy, unspecified |
| diabetes | E0941 | drug or chemical induced diabetes mellitus with neurological complications with diabetic mononeuropathy |
| diabetes | E0942 | drug or chemical induced diabetes mellitus with neurological complications with diabetic polyneuropathy |
| diabetes | E0943 | drug or chemical induced diabetes mellitus with neurological complications with diabetic autonomic (poly)neuropathy |
| diabetes | E0944 | drug or chemical induced diabetes mellitus with neurological complications with diabetic amyotrophy |
| diabetes | E0949 | drug or chemical induced diabetes mellitus with neurological complications with other diabetic neurological complication |
| diabetes | E0951 | drug or chemical induced diabetes mellitus with diabetic peripheral angiopathy without gangrene |
| diabetes | E0952 | drug or chemical induced diabetes mellitus with diabetic peripheral angiopathy with gangrene |
| diabetes | E0959 | drug or chemical induced diabetes mellitus with other circulatory complications |
| diabetes | E09610 | drug or chemical induced diabetes mellitus with diabetic neuropathic arthropathy |
| diabetes | E09618 | drug or chemical induced diabetes mellitus with other diabetic arthropathy |
| diabetes | E09620 | drug or chemical induced diabetes mellitus with diabetic dermatitis |
| diabetes | E09621 | drug or chemical induced diabetes mellitus with foot ulcer |
| diabetes | E09622 | drug or chemical induced diabetes mellitus with other skin ulcer |
| diabetes | E09628 | drug or chemical induced diabetes mellitus with other skin complications |
| diabetes | E09630 | drug or chemical induced diabetes mellitus with periodontal disease |
| diabetes | E09638 | drug or chemical induced diabetes mellitus with other oral complications |
| diabetes | E09641 | drug or chemical induced diabetes mellitus with hypoglycemia with coma |
| diabetes | E09649 | drug or chemical induced diabetes mellitus with hypoglycemia without coma |
| diabetes | E0965 | drug or chemical induced diabetes mellitus with hyperglycemia |
| diabetes | E0969 | drug or chemical induced diabetes mellitus with other specified complication |
| diabetes | E098 | drug or chemical induced diabetes mellitus with unspecified complications |
| diabetes | E099 | drug or chemical induced diabetes mellitus without complications |
| diabetes | E1010 | type 1 diabetes mellitus with ketoacidosis without coma |
| diabetes | E1011 | type 1 diabetes mellitus with ketoacidosis with coma |
| diabetes | E1021 | type 1 diabetes mellitus with diabetic nephropathy |
| diabetes | E1022 | type 1 diabetes mellitus with diabetic chronic kidney disease |
| diabetes | E1029 | type 1 diabetes mellitus with other diabetic kidney complication |
| diabetes | E10311 | type 1 diabetes mellitus with unspecified diabetic retinopathy with macular edema |
| diabetes | E10319 | type 1 diabetes mellitus with unspecified diabetic retinopathy without macular edema |
| diabetes | E10321 | type 1 diabetes mellitus with mild nonproliferative diabetic retinopathy with macular edema |
| diabetes | E103211 | type 1 diabetes mellitus with mild nonproliferative diabetic retinopathy with macular edema, right eye |
| diabetes | E103212 | type 1 diabetes mellitus with mild nonproliferative diabetic retinopathy with macular edema, left eye |
| diabetes | E103213 | type 1 diabetes mellitus with mild nonproliferative diabetic retinopathy with macular edema, bilateral |
| diabetes | E103219 | type 1 diabetes mellitus with mild nonproliferative diabetic retinopathy with macular edema, unspecified eye |
| diabetes | E10329 | type 1 diabetes mellitus with mild nonproliferative diabetic retinopathy without macular edema |
| diabetes | E103291 | type 1 diabetes mellitus with mild nonproliferative diabetic retinopathy without macular edema, right eye |
| diabetes | E103292 | type 1 diabetes mellitus with mild nonproliferative diabetic retinopathy without macular edema, left eye |
| diabetes | E103293 | type 1 diabetes mellitus with mild nonproliferative diabetic retinopathy without macular edema, bilateral |
| diabetes | E103299 | type 1 diabetes mellitus with mild nonproliferative diabetic retinopathy without macular edema, unspecified eye |
| diabetes | E10331 | type 1 diabetes mellitus with moderate nonproliferative diabetic retinopathy with macular edema |
| diabetes | E103311 | type 1 diabetes mellitus with moderate nonproliferative diabetic retinopathy with macular edema, right eye |
| diabetes | E103312 | type 1 diabetes mellitus with moderate nonproliferative diabetic retinopathy with macular edema, left eye |
| diabetes | E103313 | type 1 diabetes mellitus with moderate nonproliferative diabetic retinopathy with macular edema, bilateral |
| diabetes | E103319 | type 1 diabetes mellitus with moderate nonproliferative diabetic retinopathy with macular edema, unspecified eye |
| diabetes | E10339 | type 1 diabetes mellitus with moderate nonproliferative diabetic retinopathy without macular edema |
| diabetes | E103391 | type 1 diabetes mellitus with moderate nonproliferative diabetic retinopathy without macular edema, right eye |
| diabetes | E103392 | type 1 diabetes mellitus with moderate nonproliferative diabetic retinopathy without macular edema, left eye |
| diabetes | E103393 | type 1 diabetes mellitus with moderate nonproliferative diabetic retinopathy without macular edema, bilateral |
| diabetes | E103399 | type 1 diabetes mellitus with moderate nonproliferative diabetic retinopathy without macular edema, unspecified eye |
| diabetes | E10341 | type 1 diabetes mellitus with severe nonproliferative diabetic retinopathy with macular edema |
| diabetes | E103411 | type 1 diabetes mellitus with severe nonproliferative diabetic retinopathy with macular edema, right eye |
| diabetes | E103412 | type 1 diabetes mellitus with severe nonproliferative diabetic retinopathy with macular edema, left eye |
| diabetes | E103413 | type 1 diabetes mellitus with severe nonproliferative diabetic retinopathy with macular edema, bilateral |
| diabetes | E103419 | type 1 diabetes mellitus with severe nonproliferative diabetic retinopathy with macular edema, unspecified eye |
| diabetes | E10349 | type 1 diabetes mellitus with severe nonproliferative diabetic retinopathy without macular edema |
| diabetes | E103491 | type 1 diabetes mellitus with severe nonproliferative diabetic retinopathy without macular edema, right eye |
| diabetes | E103492 | type 1 diabetes mellitus with severe nonproliferative diabetic retinopathy without macular edema, left eye |
| diabetes | E103493 | type 1 diabetes mellitus with severe nonproliferative diabetic retinopathy without macular edema, bilateral |
| diabetes | E103499 | type 1 diabetes mellitus with severe nonproliferative diabetic retinopathy without macular edema, unspecified eye |
| diabetes | E10351 | type 1 diabetes mellitus with proliferative diabetic retinopathy with macular edema |
| diabetes | E103511 | type 1 diabetes mellitus with proliferative diabetic retinopathy with macular edema, right eye |
| diabetes | E103512 | type 1 diabetes mellitus with proliferative diabetic retinopathy with macular edema, left eye |
| diabetes | E103513 | type 1 diabetes mellitus with proliferative diabetic retinopathy with macular edema, bilateral |
| diabetes | E103519 | type 1 diabetes mellitus with proliferative diabetic retinopathy with macular edema, unspecified eye |
| diabetes | E103521 | type 1 diabetes mellitus with proliferative diabetic retinopathy with traction retinal detachment involving the macula, right eye |
| diabetes | E103522 | type 1 diabetes mellitus with proliferative diabetic retinopathy with traction retinal detachment involving the macula, left eye |
| diabetes | E103523 | type 1 diabetes mellitus with proliferative diabetic retinopathy with traction retinal detachment involving the macula, bilateral |
| diabetes | E103529 | type 1 diabetes mellitus with proliferative diabetic retinopathy with traction retinal detachment involving the macula, unspecified eye |
| diabetes | E103531 | type 1 diabetes mellitus with proliferative diabetic retinopathy with traction retinal detachment not involving the macula, right eye |
| diabetes | E103532 | type 1 diabetes mellitus with proliferative diabetic retinopathy with traction retinal detachment not involving the macula, left eye |
| diabetes | E103533 | type 1 diabetes mellitus with proliferative diabetic retinopathy with traction retinal detachment not involving the macula, bilateral |
| diabetes | E103539 | type 1 diabetes mellitus with proliferative diabetic retinopathy with traction retinal detachment not involving the macula, unspecified eye |
| diabetes | E103541 | type 1 diabetes mellitus with proliferative diabetic retinopathy with combined traction retinal detachment and rhegmatogenous retinal detachment, right eye |
| diabetes | E103542 | type 1 diabetes mellitus with proliferative diabetic retinopathy with combined traction retinal detachment and rhegmatogenous retinal detachment, left eye |
| diabetes | E103543 | type 1 diabetes mellitus with proliferative diabetic retinopathy with combined traction retinal detachment and rhegmatogenous retinal detachment, bilateral |
| diabetes | E103549 | type 1 diabetes mellitus with proliferative diabetic retinopathy with combined traction retinal detachment and rhegmatogenous retinal detachment, unspecified eye |
| diabetes | E103551 | type 1 diabetes mellitus with stable proliferative diabetic retinopathy, right eye |
| diabetes | E103552 | type 1 diabetes mellitus with stable proliferative diabetic retinopathy, left eye |
| diabetes | E103553 | type 1 diabetes mellitus with stable proliferative diabetic retinopathy, bilateral |
| diabetes | E103559 | type 1 diabetes mellitus with stable proliferative diabetic retinopathy, unspecified eye |
| diabetes | E10359 | type 1 diabetes mellitus with proliferative diabetic retinopathy without macular edema |
| diabetes | E103591 | type 1 diabetes mellitus with proliferative diabetic retinopathy without macular edema, right eye |
| diabetes | E103592 | type 1 diabetes mellitus with proliferative diabetic retinopathy without macular edema, left eye |
| diabetes | E103593 | type 1 diabetes mellitus with proliferative diabetic retinopathy without macular edema, bilateral |
| diabetes | E103599 | type 1 diabetes mellitus with proliferative diabetic retinopathy without macular edema, unspecified eye |
| diabetes | E1036 | type 1 diabetes mellitus with diabetic cataract |
| diabetes | E1037X1 | type 1 diabetes mellitus with diabetic macular edema, resolved following treatment, right eye |
| diabetes | E1037X2 | type 1 diabetes mellitus with diabetic macular edema, resolved following treatment, left eye |
| diabetes | E1037X3 | type 1 diabetes mellitus with diabetic macular edema, resolved following treatment, bilateral |
| diabetes | E1037X9 | type 1 diabetes mellitus with diabetic macular edema, resolved following treatment, unspecified eye |
| diabetes | E1039 | type 1 diabetes mellitus with other diabetic ophthalmic complication |
| diabetes | E1040 | type 1 diabetes mellitus with diabetic neuropathy, unspecified |
| diabetes | E1041 | type 1 diabetes mellitus with diabetic mononeuropathy |
| diabetes | E1042 | type 1 diabetes mellitus with diabetic polyneuropathy |
| diabetes | E1043 | type 1 diabetes mellitus with diabetic autonomic (poly)neuropathy |
| diabetes | E1044 | type 1 diabetes mellitus with diabetic amyotrophy |
| diabetes | E1049 | type 1 diabetes mellitus with other diabetic neurological complication |
| diabetes | E1051 | type 1 diabetes mellitus with diabetic peripheral angiopathy without gangrene |
| diabetes | E1052 | type 1 diabetes mellitus with diabetic peripheral angiopathy with gangrene |
| diabetes | E1059 | type 1 diabetes mellitus with other circulatory complications |
| diabetes | E10610 | type 1 diabetes mellitus with diabetic neuropathic arthropathy |
| diabetes | E10618 | type 1 diabetes mellitus with other diabetic arthropathy |
| diabetes | E10620 | type 1 diabetes mellitus with diabetic dermatitis |
| diabetes | E10621 | type 1 diabetes mellitus with foot ulcer |
| diabetes | E10622 | type 1 diabetes mellitus with other skin ulcer |
| diabetes | E10628 | type 1 diabetes mellitus with other skin complications |
| diabetes | E10630 | type 1 diabetes mellitus with periodontal disease |
| diabetes | E10638 | type 1 diabetes mellitus with other oral complications |
| diabetes | E10641 | type 1 diabetes mellitus with hypoglycemia with coma |
| diabetes | E10649 | type 1 diabetes mellitus with hypoglycemia without coma |
| diabetes | E1065 | type 1 diabetes mellitus with hyperglycemia |
| diabetes | E1069 | type 1 diabetes mellitus with other specified complication |
| diabetes | E108 | type 1 diabetes mellitus with unspecified complications |
| diabetes | E109 | type 1 diabetes mellitus without complications |
| diabetes | E1100 | type 2 diabetes mellitus with hyperosmolarity without nonketotic hyperglycemic-hyperosmolar coma (nkhhc) |
| diabetes | E1101 | type 2 diabetes mellitus with hyperosmolarity with coma |
| diabetes | E1110 | type 2 diabetes mellitus with ketoacidosis without coma |
| diabetes | E1111 | type 2 diabetes mellitus with ketoacidosis with coma |
| diabetes | E1121 | type 2 diabetes mellitus with diabetic nephropathy |
| diabetes | E1122 | type 2 diabetes mellitus with diabetic chronic kidney disease |
| diabetes | E1129 | type 2 diabetes mellitus with other diabetic kidney complication |
| diabetes | E11311 | type 2 diabetes mellitus with unspecified diabetic retinopathy with macular edema |
| diabetes | E11319 | type 2 diabetes mellitus with unspecified diabetic retinopathy without macular edema |
| diabetes | E11321 | type 2 diabetes mellitus with mild nonproliferative diabetic retinopathy with macular edema |
| diabetes | E113211 | type 2 diabetes mellitus with mild nonproliferative diabetic retinopathy with macular edema, right eye |
| diabetes | E113212 | type 2 diabetes mellitus with mild nonproliferative diabetic retinopathy with macular edema, left eye |
| diabetes | E113213 | type 2 diabetes mellitus with mild nonproliferative diabetic retinopathy with macular edema, bilateral |
| diabetes | E113219 | type 2 diabetes mellitus with mild nonproliferative diabetic retinopathy with macular edema, unspecified eye |
| diabetes | E11329 | type 2 diabetes mellitus with mild nonproliferative diabetic retinopathy without macular edema |
| diabetes | E113291 | type 2 diabetes mellitus with mild nonproliferative diabetic retinopathy without macular edema, right eye |
| diabetes | E113292 | type 2 diabetes mellitus with mild nonproliferative diabetic retinopathy without macular edema, left eye |
| diabetes | E113293 | type 2 diabetes mellitus with mild nonproliferative diabetic retinopathy without macular edema, bilateral |
| diabetes | E113299 | type 2 diabetes mellitus with mild nonproliferative diabetic retinopathy without macular edema, unspecified eye |
| diabetes | E11331 | type 2 diabetes mellitus with moderate nonproliferative diabetic retinopathy with macular edema |
| diabetes | E113311 | type 2 diabetes mellitus with moderate nonproliferative diabetic retinopathy with macular edema, right eye |
| diabetes | E113312 | type 2 diabetes mellitus with moderate nonproliferative diabetic retinopathy with macular edema, left eye |
| diabetes | E113313 | type 2 diabetes mellitus with moderate nonproliferative diabetic retinopathy with macular edema, bilateral |
| diabetes | E113319 | type 2 diabetes mellitus with moderate nonproliferative diabetic retinopathy with macular edema, unspecified eye |
| diabetes | E11339 | type 2 diabetes mellitus with moderate nonproliferative diabetic retinopathy without macular edema |
| diabetes | E113391 | type 2 diabetes mellitus with moderate nonproliferative diabetic retinopathy without macular edema, right eye |
| diabetes | E113392 | type 2 diabetes mellitus with moderate nonproliferative diabetic retinopathy without macular edema, left eye |
| diabetes | E113393 | type 2 diabetes mellitus with moderate nonproliferative diabetic retinopathy without macular edema, bilateral |
| diabetes | E113399 | type 2 diabetes mellitus with moderate nonproliferative diabetic retinopathy without macular edema, unspecified eye |
| diabetes | E11341 | type 2 diabetes mellitus with severe nonproliferative diabetic retinopathy with macular edema |
| diabetes | E113411 | type 2 diabetes mellitus with severe nonproliferative diabetic retinopathy with macular edema, right eye |
| diabetes | E113412 | type 2 diabetes mellitus with severe nonproliferative diabetic retinopathy with macular edema, left eye |
| diabetes | E113413 | type 2 diabetes mellitus with severe nonproliferative diabetic retinopathy with macular edema, bilateral |
| diabetes | E113419 | type 2 diabetes mellitus with severe nonproliferative diabetic retinopathy with macular edema, unspecified eye |
| diabetes | E11349 | type 2 diabetes mellitus with severe nonproliferative diabetic retinopathy without macular edema |
| diabetes | E113491 | type 2 diabetes mellitus with severe nonproliferative diabetic retinopathy without macular edema, right eye |
| diabetes | E113492 | type 2 diabetes mellitus with severe nonproliferative diabetic retinopathy without macular edema, left eye |
| diabetes | E113493 | type 2 diabetes mellitus with severe nonproliferative diabetic retinopathy without macular edema, bilateral |
| diabetes | E113499 | type 2 diabetes mellitus with severe nonproliferative diabetic retinopathy without macular edema, unspecified eye |
| diabetes | E11351 | type 2 diabetes mellitus with proliferative diabetic retinopathy with macular edema |
| diabetes | E113511 | type 2 diabetes mellitus with proliferative diabetic retinopathy with macular edema, right eye |
| diabetes | E113512 | type 2 diabetes mellitus with proliferative diabetic retinopathy with macular edema, left eye |
| diabetes | E113513 | type 2 diabetes mellitus with proliferative diabetic retinopathy with macular edema, bilateral |
| diabetes | E113519 | type 2 diabetes mellitus with proliferative diabetic retinopathy with macular edema, unspecified eye |
| diabetes | E113521 | type 2 diabetes mellitus with proliferative diabetic retinopathy with traction retinal detachment involving the macula, right eye |
| diabetes | E113522 | type 2 diabetes mellitus with proliferative diabetic retinopathy with traction retinal detachment involving the macula, left eye |
| diabetes | E113523 | type 2 diabetes mellitus with proliferative diabetic retinopathy with traction retinal detachment involving the macula, bilateral |
| diabetes | E113529 | type 2 diabetes mellitus with proliferative diabetic retinopathy with traction retinal detachment involving the macula, unspecified eye |
| diabetes | E113531 | type 2 diabetes mellitus with proliferative diabetic retinopathy with traction retinal detachment not involving the macula, right eye |
| diabetes | E113532 | type 2 diabetes mellitus with proliferative diabetic retinopathy with traction retinal detachment not involving the macula, left eye |
| diabetes | E113533 | type 2 diabetes mellitus with proliferative diabetic retinopathy with traction retinal detachment not involving the macula, bilateral |
| diabetes | E113539 | type 2 diabetes mellitus with proliferative diabetic retinopathy with traction retinal detachment not involving the macula, unspecified eye |
| diabetes | E113541 | type 2 diabetes mellitus with proliferative diabetic retinopathy with combined traction retinal detachment and rhegmatogenous retinal detachment, right eye |
| diabetes | E113542 | type 2 diabetes mellitus with proliferative diabetic retinopathy with combined traction retinal detachment and rhegmatogenous retinal detachment, left eye |
| diabetes | E113543 | type 2 diabetes mellitus with proliferative diabetic retinopathy with combined traction retinal detachment and rhegmatogenous retinal detachment, bilateral |
| diabetes | E113549 | type 2 diabetes mellitus with proliferative diabetic retinopathy with combined traction retinal detachment and rhegmatogenous retinal detachment, unspecified eye |
| diabetes | E113551 | type 2 diabetes mellitus with stable proliferative diabetic retinopathy, right eye |
| diabetes | E113552 | type 2 diabetes mellitus with stable proliferative diabetic retinopathy, left eye |
| diabetes | E113553 | type 2 diabetes mellitus with stable proliferative diabetic retinopathy, bilateral |
| diabetes | E113559 | type 2 diabetes mellitus with stable proliferative diabetic retinopathy, unspecified eye |
| diabetes | E11359 | type 2 diabetes mellitus with proliferative diabetic retinopathy without macular edema |
| diabetes | E113591 | type 2 diabetes mellitus with proliferative diabetic retinopathy without macular edema, right eye |
| diabetes | E113592 | type 2 diabetes mellitus with proliferative diabetic retinopathy without macular edema, left eye |
| diabetes | E113593 | type 2 diabetes mellitus with proliferative diabetic retinopathy without macular edema, bilateral |
| diabetes | E113599 | type 2 diabetes mellitus with proliferative diabetic retinopathy without macular edema, unspecified eye |
| diabetes | E1136 | type 2 diabetes mellitus with diabetic cataract |
| diabetes | E1137X1 | type 2 diabetes mellitus with diabetic macular edema, resolved following treatment, right eye |
| diabetes | E1137X2 | type 2 diabetes mellitus with diabetic macular edema, resolved following treatment, left eye |
| diabetes | E1137X3 | type 2 diabetes mellitus with diabetic macular edema, resolved following treatment, bilateral |
| diabetes | E1137X9 | type 2 diabetes mellitus with diabetic macular edema, resolved following treatment, unspecified eye |
| diabetes | E1139 | type 2 diabetes mellitus with other diabetic ophthalmic complication |
| diabetes | E1140 | type 2 diabetes mellitus with diabetic neuropathy, unspecified |
| diabetes | E1141 | type 2 diabetes mellitus with diabetic mononeuropathy |
| diabetes | E1142 | type 2 diabetes mellitus with diabetic polyneuropathy |
| diabetes | E1143 | type 2 diabetes mellitus with diabetic autonomic (poly)neuropathy |
| diabetes | E1144 | type 2 diabetes mellitus with diabetic amyotrophy |
| diabetes | E1149 | type 2 diabetes mellitus with other diabetic neurological complication |
| diabetes | E1151 | type 2 diabetes mellitus with diabetic peripheral angiopathy without gangrene |
| diabetes | E1152 | type 2 diabetes mellitus with diabetic peripheral angiopathy with gangrene |
| diabetes | E1159 | type 2 diabetes mellitus with other circulatory complications |
| diabetes | E11610 | type 2 diabetes mellitus with diabetic neuropathic arthropathy |
| diabetes | E11618 | type 2 diabetes mellitus with other diabetic arthropathy |
| diabetes | E11620 | type 2 diabetes mellitus with diabetic dermatitis |
| diabetes | E11621 | type 2 diabetes mellitus with foot ulcer |
| diabetes | E11622 | type 2 diabetes mellitus with other skin ulcer |
| diabetes | E11628 | type 2 diabetes mellitus with other skin complications |
| diabetes | E11630 | type 2 diabetes mellitus with periodontal disease |
| diabetes | E11638 | type 2 diabetes mellitus with other oral complications |
| diabetes | E11641 | type 2 diabetes mellitus with hypoglycemia with coma |
| diabetes | E11649 | type 2 diabetes mellitus with hypoglycemia without coma |
| diabetes | E1165 | type 2 diabetes mellitus with hyperglycemia |
| diabetes | E1169 | type 2 diabetes mellitus with other specified complication |
| diabetes | E118 | type 2 diabetes mellitus with unspecified complications |
| diabetes | E119 | type 2 diabetes mellitus without complications |
| diabetes | E1300 | other specified diabetes mellitus with hyperosmolarity without nonketotic hyperglycemic-hyperosmolar coma (nkhhc) |
| diabetes | E1301 | other specified diabetes mellitus with hyperosmolarity with coma |
| diabetes | E1310 | other specified diabetes mellitus with ketoacidosis without coma |
| diabetes | E1311 | other specified diabetes mellitus with ketoacidosis with coma |
| diabetes | E1321 | other specified diabetes mellitus with diabetic nephropathy |
| diabetes | E1322 | other specified diabetes mellitus with diabetic chronic kidney disease |
| diabetes | E1329 | other specified diabetes mellitus with other diabetic kidney complication |
| diabetes | E13311 | other specified diabetes mellitus with unspecified diabetic retinopathy with macular edema |
| diabetes | E13319 | other specified diabetes mellitus with unspecified diabetic retinopathy without macular edema |
| diabetes | E13321 | other specified diabetes mellitus with mild nonproliferative diabetic retinopathy with macular edema |
| diabetes | E133211 | other specified diabetes mellitus with mild nonproliferative diabetic retinopathy with macular edema, right eye |
| diabetes | E133212 | other specified diabetes mellitus with mild nonproliferative diabetic retinopathy with macular edema, left eye |
| diabetes | E133213 | other specified diabetes mellitus with mild nonproliferative diabetic retinopathy with macular edema, bilateral |
| diabetes | E133219 | other specified diabetes mellitus with mild nonproliferative diabetic retinopathy with macular edema, unspecified eye |
| diabetes | E13329 | other specified diabetes mellitus with mild nonproliferative diabetic retinopathy without macular edema |
| diabetes | E133291 | other specified diabetes mellitus with mild nonproliferative diabetic retinopathy without macular edema, right eye |
| diabetes | E133292 | other specified diabetes mellitus with mild nonproliferative diabetic retinopathy without macular edema, left eye |
| diabetes | E133293 | other specified diabetes mellitus with mild nonproliferative diabetic retinopathy without macular edema, bilateral |
| diabetes | E133299 | other specified diabetes mellitus with mild nonproliferative diabetic retinopathy without macular edema, unspecified eye |
| diabetes | E13331 | other specified diabetes mellitus with moderate nonproliferative diabetic retinopathy with macular edema |
| diabetes | E133311 | other specified diabetes mellitus with moderate nonproliferative diabetic retinopathy with macular edema, right eye |
| diabetes | E133312 | other specified diabetes mellitus with moderate nonproliferative diabetic retinopathy with macular edema, left eye |
| diabetes | E133313 | other specified diabetes mellitus with moderate nonproliferative diabetic retinopathy with macular edema, bilateral |
| diabetes | E133319 | other specified diabetes mellitus with moderate nonproliferative diabetic retinopathy with macular edema, unspecified eye |
| diabetes | E13339 | other specified diabetes mellitus with moderate nonproliferative diabetic retinopathy without macular edema |
| diabetes | E133391 | other specified diabetes mellitus with moderate nonproliferative diabetic retinopathy without macular edema, right eye |
| diabetes | E133392 | other specified diabetes mellitus with moderate nonproliferative diabetic retinopathy without macular edema, left eye |
| diabetes | E133393 | other specified diabetes mellitus with moderate nonproliferative diabetic retinopathy without macular edema, bilateral |
| diabetes | E133399 | other specified diabetes mellitus with moderate nonproliferative diabetic retinopathy without macular edema, unspecified eye |
| diabetes | E13341 | other specified diabetes mellitus with severe nonproliferative diabetic retinopathy with macular edema |
| diabetes | E133411 | other specified diabetes mellitus with severe nonproliferative diabetic retinopathy with macular edema, right eye |
| diabetes | E133412 | other specified diabetes mellitus with severe nonproliferative diabetic retinopathy with macular edema, left eye |
| diabetes | E133413 | other specified diabetes mellitus with severe nonproliferative diabetic retinopathy with macular edema, bilateral |
| diabetes | E133419 | other specified diabetes mellitus with severe nonproliferative diabetic retinopathy with macular edema, unspecified eye |
| diabetes | E13349 | other specified diabetes mellitus with severe nonproliferative diabetic retinopathy without macular edema |
| diabetes | E133491 | other specified diabetes mellitus with severe nonproliferative diabetic retinopathy without macular edema, right eye |
| diabetes | E133492 | other specified diabetes mellitus with severe nonproliferative diabetic retinopathy without macular edema, left eye |
| diabetes | E133493 | other specified diabetes mellitus with severe nonproliferative diabetic retinopathy without macular edema, bilateral |
| diabetes | E133499 | other specified diabetes mellitus with severe nonproliferative diabetic retinopathy without macular edema, unspecified eye |
| diabetes | E13351 | other specified diabetes mellitus with proliferative diabetic retinopathy with macular edema |
| diabetes | E133511 | other specified diabetes mellitus with proliferative diabetic retinopathy with macular edema, right eye |
| diabetes | E133512 | other specified diabetes mellitus with proliferative diabetic retinopathy with macular edema, left eye |
| diabetes | E133513 | other specified diabetes mellitus with proliferative diabetic retinopathy with macular edema, bilateral |
| diabetes | E133519 | other specified diabetes mellitus with proliferative diabetic retinopathy with macular edema, unspecified eye |
| diabetes | E133521 | other specified diabetes mellitus with proliferative diabetic retinopathy with traction retinal detachment involving the macula, right eye |
| diabetes | E133522 | other specified diabetes mellitus with proliferative diabetic retinopathy with traction retinal detachment involving the macula, left eye |
| diabetes | E133523 | other specified diabetes mellitus with proliferative diabetic retinopathy with traction retinal detachment involving the macula, bilateral |
| diabetes | E133529 | other specified diabetes mellitus with proliferative diabetic retinopathy with traction retinal detachment involving the macula, unspecified eye |
| diabetes | E133531 | other specified diabetes mellitus with proliferative diabetic retinopathy with traction retinal detachment not involving the macula, right eye |
| diabetes | E133532 | other specified diabetes mellitus with proliferative diabetic retinopathy with traction retinal detachment not involving the macula, left eye |
| diabetes | E133533 | other specified diabetes mellitus with proliferative diabetic retinopathy with traction retinal detachment not involving the macula, bilateral |
| diabetes | E133539 | other specified diabetes mellitus with proliferative diabetic retinopathy with traction retinal detachment not involving the macula, unspecified eye |
| diabetes | E133541 | other specified diabetes mellitus with proliferative diabetic retinopathy with combined traction retinal detachment and rhegmatogenous retinal detachment, right eye |
| diabetes | E133542 | other specified diabetes mellitus with proliferative diabetic retinopathy with combined traction retinal detachment and rhegmatogenous retinal detachment, left eye |
| diabetes | E133543 | other specified diabetes mellitus with proliferative diabetic retinopathy with combined traction retinal detachment and rhegmatogenous retinal detachment, bilateral |
| diabetes | E133549 | other specified diabetes mellitus with proliferative diabetic retinopathy with combined traction retinal detachment and rhegmatogenous retinal detachment, unspecified eye |
| diabetes | E133551 | other specified diabetes mellitus with stable proliferative diabetic retinopathy, right eye |
| diabetes | E133552 | other specified diabetes mellitus with stable proliferative diabetic retinopathy, left eye |
| diabetes | E133553 | other specified diabetes mellitus with stable proliferative diabetic retinopathy, bilateral |
| diabetes | E133559 | other specified diabetes mellitus with stable proliferative diabetic retinopathy, unspecified eye |
| diabetes | E13359 | other specified diabetes mellitus with proliferative diabetic retinopathy without macular edema |
| diabetes | E133591 | other specified diabetes mellitus with proliferative diabetic retinopathy without macular edema, right eye |
| diabetes | E133592 | other specified diabetes mellitus with proliferative diabetic retinopathy without macular edema, left eye |
| diabetes | E133593 | other specified diabetes mellitus with proliferative diabetic retinopathy without macular edema, bilateral |
| diabetes | E133599 | other specified diabetes mellitus with proliferative diabetic retinopathy without macular edema, unspecified eye |
| diabetes | E1336 | other specified diabetes mellitus with diabetic cataract |
| diabetes | E1337X1 | other specified diabetes mellitus with diabetic macular edema, resolved following treatment, right eye |
| diabetes | E1337X2 | other specified diabetes mellitus with diabetic macular edema, resolved following treatment, left eye |
| diabetes | E1337X3 | other specified diabetes mellitus with diabetic macular edema, resolved following treatment, bilateral |
| diabetes | E1337X9 | other specified diabetes mellitus with diabetic macular edema, resolved following treatment, unspecified eye |
| diabetes | E1339 | other specified diabetes mellitus with other diabetic ophthalmic complication |
| diabetes | E1340 | other specified diabetes mellitus with diabetic neuropathy, unspecified |
| diabetes | E1341 | other specified diabetes mellitus with diabetic mononeuropathy |
| diabetes | E1342 | other specified diabetes mellitus with diabetic polyneuropathy |
| diabetes | E1343 | other specified diabetes mellitus with diabetic autonomic (poly)neuropathy |
| diabetes | E1344 | other specified diabetes mellitus with diabetic amyotrophy |
| diabetes | E1349 | other specified diabetes mellitus with other diabetic neurological complication |
| diabetes | E1351 | other specified diabetes mellitus with diabetic peripheral angiopathy without gangrene |
| diabetes | E1352 | other specified diabetes mellitus with diabetic peripheral angiopathy with gangrene |
| diabetes | E1359 | other specified diabetes mellitus with other circulatory complications |
| diabetes | E13610 | other specified diabetes mellitus with diabetic neuropathic arthropathy |
| diabetes | E13618 | other specified diabetes mellitus with other diabetic arthropathy |
| diabetes | E13620 | other specified diabetes mellitus with diabetic dermatitis |
| diabetes | E13621 | other specified diabetes mellitus with foot ulcer |
| diabetes | E13622 | other specified diabetes mellitus with other skin ulcer |
| diabetes | E13628 | other specified diabetes mellitus with other skin complications |
| diabetes | E13630 | other specified diabetes mellitus with periodontal disease |
| diabetes | E13638 | other specified diabetes mellitus with other oral complications |
| diabetes | E13641 | other specified diabetes mellitus with hypoglycemia with coma |
| diabetes | E13649 | other specified diabetes mellitus with hypoglycemia without coma |
| diabetes | E1365 | other specified diabetes mellitus with hyperglycemia |
| diabetes | E1369 | other specified diabetes mellitus with other specified complication |
| diabetes | E138 | other specified diabetes mellitus with unspecified complications |
| diabetes | E139 | other specified diabetes mellitus without complications |
| diabetes | V5867 | long-term (current) use of insulin |
| diabetes | Z794 | long term (current) use of insulin |
| diabetes | Z7984 | long term (current) use of oral hypoglycemic drugs |

# Appendix B: Procedure Definitions

| **Group** | **HCPCS Code or Range** | **Description** |
| --- | --- | --- |
| any imaging | 93880 | duplex scan of extracranial arteries; complete bilateral study |
| any imaging | 93882 | duplex scan of extracranial arteries; unilateral or limited study |
| any imaging | 70010 - 76499 | diagnostic radiology |
| any imaging | 76506 - 76999 | diagnostic ultrasound |
| any imaging | 77071 - 77086 | bone / joint studies |
| any imaging | 78012 - 79999 | nuclear medicine |
| any oncology | 77261 - 77799 | radiation oncology |
| any oncology | J8501 - J8999 | oral anti-cancer drugs |
| any oncology | J9000 - J9999 | chemotherapy drugs |
| chest imaging | 76604 | ultrasound chest exam |
| chest imaging | 78414 | determination of central c-v hemodynamics |
| chest imaging | 78428 | cardiac shunt imaging |
| chest imaging | 75557 - 75574 | cardiac magnetic resonance imaging studies |
| chest imaging | 78451 - 78496 | myocardial perfusion imaging studies |
| neuroimaging | 70250 | radiologic examination, skull; less than 4 views |
| neuroimaging | 70260 | radiologic examination, skull; complete, minimum of 4 views |
| neuroimaging | 70450 | computed tomography, head or brain; without contrast material |
| neuroimaging | 70460 | computed tomography, head or brain; with contrast material(s) |
| neuroimaging | 70470 | computed tomography, head or brain; without contrast material, followed by contrast material(s) and further sections |
| neuroimaging | 70496 | computed tomographic angiography, head, with contrast material(s), including noncontrast images, if performed, and image postprocessing |
| neuroimaging | 70498 | computed tomographic angiography, neck, with contrast material(s), including noncontrast images, if performed, and image postprocessing |
| neuroimaging | 70544 | magnetic resonance angiography, head; without contrast material(s) |
| neuroimaging | 70545 | magnetic resonance angiography, head; with contrast material(s) |
| neuroimaging | 70546 | magnetic resonance angiography, head; without contrast material(s), followed by contrast material(s) and further sequences |
| neuroimaging | 70547 | magnetic resonance angiography, neck; without contrast material(s) |
| neuroimaging | 70548 | magnetic resonance angiography, neck; with contrast material(s) |
| neuroimaging | 70549 | magnetic resonance angiography, neck; without contrast material(s), followed by contrast material(s) and further sequences |
| neuroimaging | 70551 | magnetic resonance (eg, proton) imaging, brain (including brain stem); without contrast material |
| neuroimaging | 70552 | magnetic resonance (eg, proton) imaging, brain (including brain stem); with contrast material(s) |
| neuroimaging | 70553 | magnetic resonance (eg, proton) imaging, brain (including brain stem); without contrast material, followed by contrast material(s) and further sequences |
| neuroimaging | 70554 | magnetic resonance imaging, brain, functional mri; including test selection and administration of repetitive body part movement and/or visual stimulation, not requiring physician or psychologist administration |
| neuroimaging | 70555 | magnetic resonance imaging, brain, functional mri; requiring physician or psychologist administration of entire neurofunctional testing |
| neuroimaging | 76506 | echoencephalography, real time with image documentation (gray scale) (for determination of ventricular size, delineation of cerebral contents, and detection of fluid masses or other intracranial abnormalities), including a-mode encephalography as secondary component where indicated |
| neuroimaging | 78605 | imaging of brain with blood flow, minimum of 4 static views |
| neuroimaging | 78606 | brain imaging, minimum 4 static views; with vascular flow |
| neuroimaging | 78607 | brain imaging, tomographic (spect) |
| neuroimaging | 78608 | brain imaging, positron emission tomography (pet); metabolic evaluation |
| neuroimaging | 78609 | brain imaging, positron emission tomography (pet); perfusion evaluation |
| neuroimaging | 78610 | brain imaging, vascular flow only |
| neuroimaging | 93880 | duplex scan of extracranial arteries; complete bilateral study |
| neuroimaging | 93882 | duplex scan of extracranial arteries; unilateral or limited study |
